# A novel DNA sequence motif in human and mouse genomes

**DOI:** 10.1038/srep10444

**Published:** 2015-05-20

**Authors:** Shilu Zhang, Fang Du, Hongkai Ji

**Affiliations:** 1 Department of Biostatistics, Johns Hopkins Bloomberg School of Public Health, 615 North Wolfe Street, Baltimore, Maryland 21205, USA

## Abstract

We report a novel DNA sequence motif in human and mouse genomes. This motif has several interesting features indicating that it is highly likely to be an unknown functional sequence element. The motif is highly enriched in promoter regions. Locations of the motif sites in the genome have strong tendency to be clustered together. Motif sites are associated with increased phylogenetic conservation as well as elevated DNase I hypersensitivity (DHS) in ENCODE cell lines. Clustered motif sites are found in promoter regions of a substantial fraction of the protein-coding genes in the genome. All together, these indicate that the motif may have important functions associated with a large number of genes.

Functional sequence elements (e.g., transcription factor binding sites) in a genome are often associated with specific DNA sequence patterns called motifs. Different types of functional elements can have different motifs. Building a vocabulary of functional DNA motifs is important for understanding how information on various biological processes is encoded in the genome. A large number of motifs have already been documented in databases^1^,^2^,^3^. Many of them are contributed by efforts that try to search for motifs systematically using either computational^4^,^5^ or experimental approaches^6^,^7^. However, since each motif search approach may have its own limitations that prevent it from discovering all functional motifs in the genome, our current motif dictionary is still far from complete. In order to build a comprehensive motif dictionary, it is important to supplement the existing motif catalog by motifs discovered sporadically from various studies.

In this study, we report a motif discovered during our routine analysis of genome-wide chromatin immunoprecipitation data for transcription factors. The motif was originally found from a collection of mouse GLI3 binding sites (Fig. 1, see Methods). This motif does not match with any existing motif in TRANSFAC^1^, UniPROBE^2^ and JASPAR^3^, and we do not have a clear clue on its function in GLI3-mediated gene regulation. However, the motif has several striking features: (1) its motif sites in the genome show strong preference to be clustered, (2) the motif is highly enriched in promoter regions, and (3) the motif sites are associated with increased phylogenetic conservation and DNase I hypersensitivity (DHS) compared to control genomic sites and control motifs. Since these features are often associated with functional *cis*-regulatory elements such as transcription factor binding sites but less so with random genomic sites^8^,^9^,^10^ , they indicate that the motif is very likely to be part of the functional regulatory vocabulary in human and mouse genomes. A substantial fraction of the protein-coding genes have at least one cluster of these motif sites in their promoters. This suggests that the motif may represent an important regulatory signal involved in regulating a large number of genes. Given the strong computational evidence supporting the motif being an important functional element, we believe that it is worthwhile to document this motif and hope that experimental biologists can establish its exact function experimentally in the future.

## Results

### Discovery of the new motif

The motif was discovered when we ran *de novo* motif discovery on a collection of GLI3 binding regions obtained by intersecting a genome-wide ChIP-chip experiment^11^ with a list of genes predominately expressed in sonic hedgehog (Shh)-responsive regions of E10.5 and E11.5 mouse embryos (see Supplementary Table S1 for genomic coordinates of these regions). GLI3 is an important transcription factor in the sonic hedgehog signaling pathway. Our motif analysis identified 6 motifs enriched ≥2 folds in the analyzed GLI3 binding regions compared to matched genomic control regions chosen to match the genomic distribution of GLI3 binding sites (Methods, Supplementary Figure S1). These include the new motif described here, two known motifs GLI and SP1, and three other unknown motifs labeled as “*de novo* class 1” in Supplementary Figure S1. Comparing the information content of the new motif to 525 known motifs in TRANSFAC shows that 70.3% of the known motifs had lower total information content compared to the new motif, and 11.6% of the known motifs had lower per-nucleotide information content compared to the new motif (Methods, Supplementary Figure S2). Thus, the sequence specificity of the new motif is within the typical range for known motifs. Although the per-nucleotide information content of the new motif is not very high, it is compensated by the length of the motif to confer relatively strong total sequence specificity. We did not find a clear clue on the potential function of the new motif in GLI3-mediated gene regulation. However, mapping the motif to both mouse and human genomes revealed a number of striking characteristics as detailed below.

### Locations of motif sites show non-random clustering

**Figure 2:**
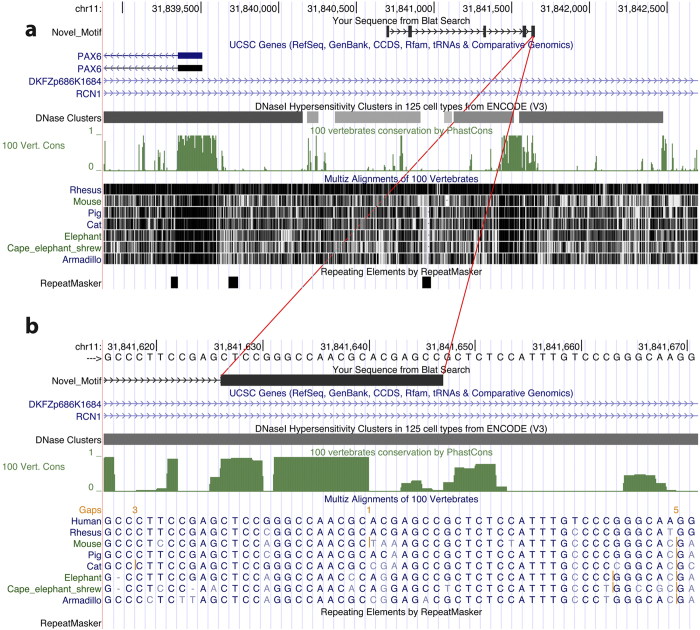
An example of clustered motif sites. (**a**) Five motif sites indicated by the thick black blocks in the “Novel_Motif” track were found near the human *PAX6* gene. (**b**) Zoomed-in plot for one motif site.

Mapping the position-specific probability matrix of the motif (Supplementary Table S2) to the human genome (hg19) using CisGenome (likelihood ratio (LR) cutoff=1000) yielded 205,283 motif sites (Methods, Table 1). Visual inspection of the motif site locations indicates that they show strong preference to be clustered together (Fig. 2). In order to systematically evaluate this clustering tendency, motif sites separated by ≤500 bp were grouped into clusters. After eliminating clusters with only one motif site, 23,152 motif site clusters were identified in the human genome. Based on our definition of cluster, each of these clusters has≥2 motif sites. Every motif site within a cluster is separated by ≤500 bp from another site in the same cluster. Any two motif sites separated by ≤500 bp belong to the same cluster. In total, these 23,152 clusters covered 89,915 (43.80% of 205,283) motif sites (Table 1). As a comparison, we randomly sampled 205,283 sites from non-repeat regions in the genome. Applying the same clustering criteria to these random genomic sites only resulted in 9,846 clusters, covering 20,223 (9.85% of 205,283) sites. Therefore, locations of the new motif in the genome showed significant clustering tendency (43.80% vs. 9.85%, Z-test p-value<1e^–15^).

To further evaluate this clustering tendency, we compared the new motif to a number of known, simulated, or computationally discovered motifs, including (i) eight known transcription factor binding motifs – GLI, CTCF, NRSF, FOXA1, MYC, Estrogen Receptor (ER), the OCT4-SOX2 composite motif, and SP1 (“known motifs”, Supplementary Figure S3), (ii) 500 matching control motifs generated by permuting the probability matrix of the new motif (“permuted motifs”, Methods), (iii) three motifs obtained from the GLI3 ChIP-chip analysis by *de novo* motif discovery which were enriched≥2 folds in GLI3 binding regions compared to matched genomic control regions (“*de novo* class 1” in Supplementary Figure S1; GLI and SP1 were excluded since they were analyzed as known motifs), and (iv) three additional motifs obtained from the GLI3 ChIP-chip analysis when applying a relaxed enrichment cutoff (≥1.2 folds instead of ≥2 folds) to filter motifs (“*de novo* class 2” in Supplementary Figure S1). These motifs are collectively called control motifs. Each control motif (except NRSF) was mapped to the genome to produce the same number (i.e., 205,283) of motif sites as the new motif. For NRSF, we only obtained 18,974 motif sites at the minimal likelihood ratio cutoff (LR ≥ 100) recommended by CisGenome, and hence the analysis of NRSF was based on these 18,974 motif sites. For all control motifs, the mapped motif sites were used to define motif site clusters in the same way as the new motif. On average, the control motifs produced fewer clusters and fewer clustered motif sites compared to the new motif (Table 1). Only 1.2% of the 500 permuted motifs, 1 (SP1) of the 8 known motifs, 1 of the *de novo* class 1 motifs, and 0 of the *de novo* class 2 motifs produced≥23,152 clusters. Only 1.8% of the 500 permuted motifs, 0 of the 8 known motifs, 1 of the *de novo* class 1 motifs, and 0 of the *de novo* class 2 motifs produced ≥89,915 clustered motif sites. Table 1 also shows that the known motifs on average had stronger clustering tendency compared to random genomic sites, while the clustering tendency of the new motif was stronger than most of the known motifs. Thus, the clustering tendency of the new motif was non-trivial.

Similar results were obtained when the same analyses were applied to the mouse genome (Table 1, Methods). Together, these results show that in both human and mouse genomes, the motif sites exhibit strong clustering tendency.

### Phylogenetic conservation of motif sites

Since functional sequence elements tend to be conserved across species due to the pressure of negative selection, we examined whether the motif sites were phylogenetically conserved using the phastCons scores^12^ downloaded from the UCSC genome browser. These phastCons scores were computed based on multiple alignments of genome sequences of different species^12^. Vast amounts of previous studies have shown that despite potential alignment uncertainties, these scores can be used to reliably characterize the average conservation level of a large number of genomic sites, such as average conservation of all binding sites of a transcription factor^13^,^14^,^15^ (see Supplementary Figure S4 for examples). This is because variability of the conservation analysis due to alignment uncertainties at individual sites is greatly reduced by averaging across a large number of sites.

**Figure 3:**
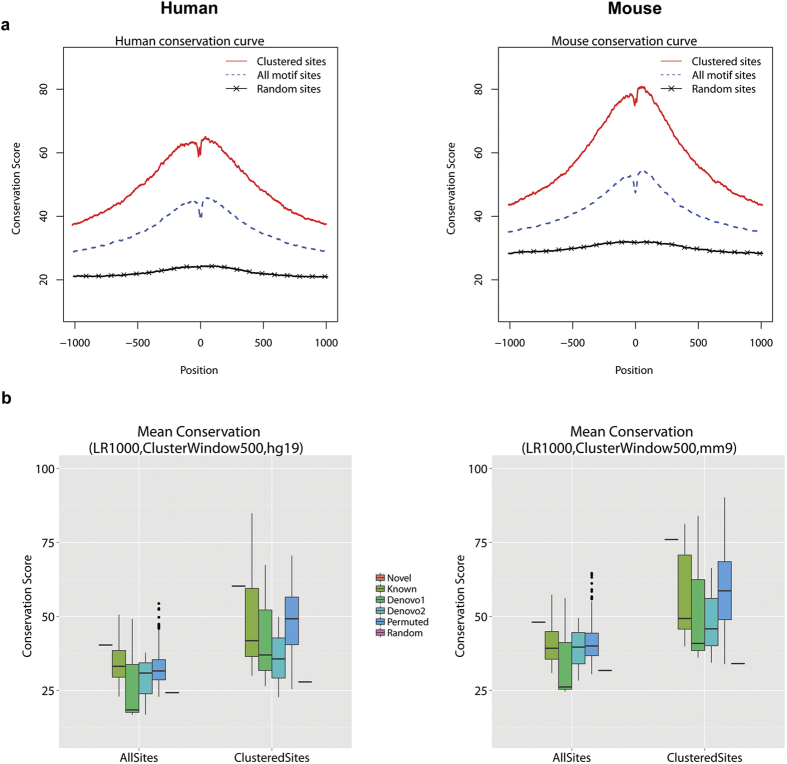
Phylogenetic conservation of the new motif. (**a**) Mean phastCons conservation scores for the motif and flanking positions in human and mouse genomes. Position 0 corresponds to the motif center. Within each genome, the scores at each position were averaged across all loci in each of the three categories: clustered motif sites, all motif sites, and random genomic sites. (**b**) Comparison of overall conservation at motif sites in human (left) and mouse (right) between the new motif and different types of control motifs. For each species, conservation was compared based on both all motif sites (“AllSites”) and clustered motif sites (“ClusteredSites”). Within each motif site category, the boxplots from left to right show the conservation of the novel motif (“Novel”), eight known motifs (“Known”), three *de novo* class 1 motifs (“Denovo1”), three *de novo* class 2 motifs (“Denovo2”), 500 permuted motifs (“Permuted”), and random genomic controls (“Random”). For each motif, the overall conservation was the average phastCons score across all motif sites. For each control motif type, the boxplot shows the distribution of overall conservation across all motifs. The new motif was mapped to genome using likelihood ratio≥1000. Motif site clusters were defined using window size=500 bp.

Using phastCons scores, we studied the average conservation profile of the motif and its flanking sequences. Each motif site was extended 1000 bp towards both ends, and the mean phastCons conservation scores for the motif and its flanking sequences averaged across all motif sites were shown in Fig. 3a. For both human and mouse, the cross-species conservation decreased when one moved away from the motif sites. The figure also shows that on average the motif sites were more conserved than random genomic sites, and the conservation further increased for clustered motif sites.

Comparing the new motif with the control motifs, not all known motifs were more conserved than the new motif, but the known motifs generally were more conserved than random genomic sites (Supplementary Figure S5). On average, the 500 permuted motifs, *de novo* class 1, and *de novo* class 2 motifs were also less conserved than the new motif (Supplementary Figure S6). Figure 3b compares the overall conservation between the new motif and different categories of control motifs. Here the overall conservation of a motif was defined as the average conservation score of all its motif sites, where the conservation score of a motif site was defined as the average phastCons score across all positions within the motif site. This analysis shows that 7.2% of the 500 permuted motifs, 2 (CTCF, NRSF) of the 8 known motifs, 1 of the *de novo* class 1 motifs, and 0 of the *de novo* class 2 motifs had the same or higher level of conservation at their motif sites compared to the new motif. Therefore, the overall conservation level of the new motif is higher than the majority of the control motifs. Importantly, only 1.2% of the 500 permuted motifs, 0 of the 8 known motifs, 1 of the *de novo* class 1 motifs, and 0 of the *de novo* class 2 motifs were as extreme as or more extreme than the new motif in terms of both clustering tendency and phylogenetic conservation (i.e., produced more motif site clusters (≥23,152) than the new motif and at the same time had the same or higher level of overall conservation at their motif sites compared to the new motif). A similar conservation analysis was also performed on the clustered motif sites (Fig. 3b). This analysis shows that 11.8% of the 500 permuted motifs, 2 (CTCF, NRSF) of the 8 known motifs, 1 of the *de novo* class 1 motifs, and 0 of the *de novo* class 2 motifs showed the same or higher level of conservation at their clustered motif sites compared to the new motif. Furthermore, only 1.2% of the 500 permuted motifs, 0 of the 8 known motifs, 1 of the *de novo* class 1 motifs, and 0 of the *de novo* class 2 motifs produced more motif site clusters (≥23,152) than the new motif and at the same time had the same or higher level of overall conservation at their clustered motif sites compared to the new motif.

### The motif is highly enriched in promoters

Analyses of motif site locations using CisGenome shows that the new motif is highly enriched in promoter regions compared to random genomic sites (Supplementary Table S3). Among the 205,283 motif sites of the new motif, 11.28% (23,159) were located within 1kb 5’-upstream of a transcription start site (TSS). By contrast, only 1.24% (2,550/205,283) of the random genomic sites were located within 1kb upstream of TSS (Table 1). Thus the motif is enriched 9.1 folds in promoter regions (defined as 1kb upstream of TSS) compared to random expectation (One sided p-value of Z-test for comparing the two proportions<1e^–15^). From another perspective, among every 9.1 motif sites found in promoters, only 1 is expected to occur by chance if the 205,283 sites were randomly distributed in the non-repeat part of the genome. This translates into an estimated false discovery rate (FDR) of 1/9.1 = 11.0%.

The promoter enrichment was more pronounced for clustered motif sites. Among the 23,152 motif site clusters of the new motif, 15.71% were located within 1kb upstream of TSS regions, representing an enrichment of 10.4 folds compared to only 1.51% of the 9,846 clusters of random genomic sites being associated with 1kb TSS upstream regions (Table 1, Z-test p-value<1e^–15^). To put it in other words, only 1 out of every 10.4 motif site clusters found in promoters is expected to occur by chance, translating into an FDR of 9.6% for detecting non-random clusters in promoter regions. Among the 89,915 clustered motif sites, 22.44% were located within 1kb upstream of TSS regions. As a comparison, only 1.53% of 20,223 randomly clustered sites were found within TSS upstream 1kb regions (Table 1, fold enrichment=14.7, Z-test p-value<1e^–15^).

**Figure 4:**
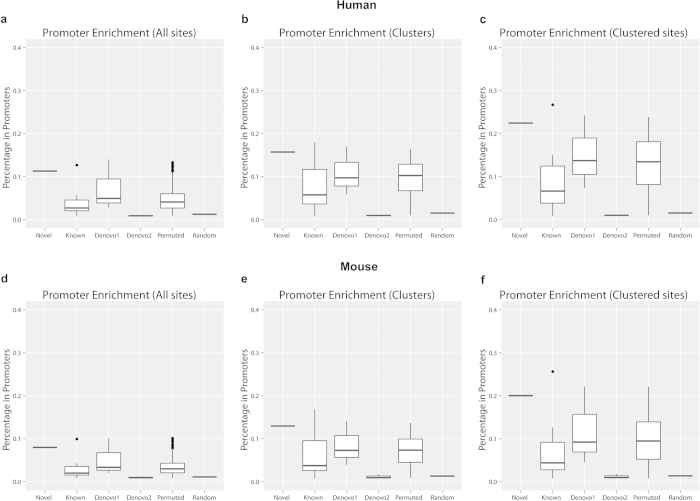
Promoter enrichment of the new motif, different classes of control motifs, and random genomic sites. For each motif, the percentages of motif sites, motif site clusters, and clustered motif sites that were in promoter regions were computed. Distributions of these percentages across all motifs in each motif category were then shown using boxplots. Six categories shown in the figure include the novel motif (“Novel”), eight known motifs (“Known”), three *de novo* class 1 motifs (“Denovo1”), three *de novo* class 2 motifs (“Denovo2”), 500 permuted motifs (“Permuted”), and random genomic control sites (“Random”). (**a**) Promoter enrichment of all human motif sites. (**b**) Promoter enrichment of all human motif site clusters. (**c**) Promoter enrichment of all human clustered motif sites. (**d**)-(**f**) are similar plots for mouse. The new motif was mapped to genome using likelihood ratio≥1000. Promoter was defined as the 1kb region upstream of transcription start site. Motif site clusters were defined using window size=500 bp.

Analyses of the control motifs further confirmed the promoter enrichment of the new motif (Table 1, Fig. 4a). Only 1.8% of the 500 permuted motifs showed the same or higher level of promoter enrichment of their motif sites compared to the new motif. The majority of the 8 known motifs were more enriched in promoters compared to random genomic sites. However, only SP1 had higher promoter enrichment compared to the new motif (Table 1). Whereas 1 of the three *de novo* class 1 motifs had higher promoter enrichment, none of the *de novo* class 2 motifs was more enriched in promoter regions than the new motif (Fig. 4a). Similar results were obtained for the promoter enrichments of clusters and clustered motif sites (Table 1, Fig. 4b,c).

Repeating the same analysis on the mouse genome (mm9) produced essentially the same results (Table 1, Fig. 4d–f). Together, these analyses demonstrate that the new motif is highly enriched in promoter regions.

### Motif sites are associated with increased DNase I hypersensitivity

**Figure 5:**
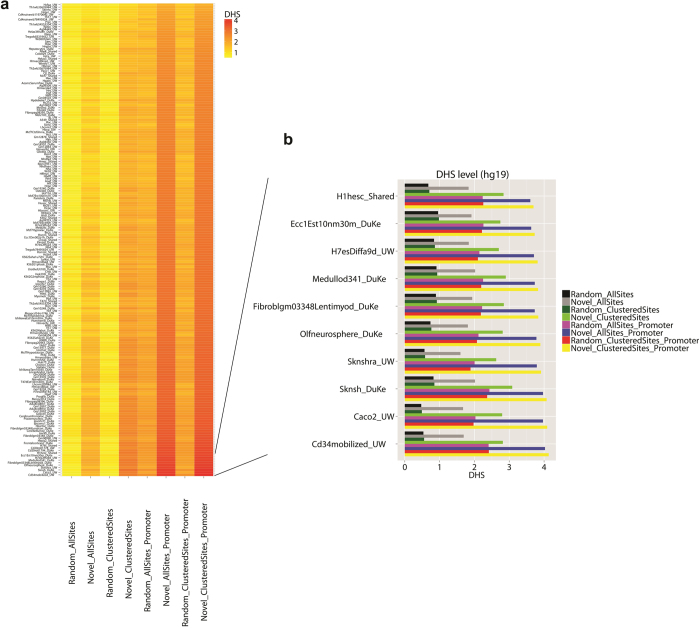
DNase I hypersensitivity for different motif site categories in 193 human ENCODE cell lines. The motif site categories shown in the figure include all motif sites (Novel_AllSites), random control sites (Random_AllSites), clustered motif sites (Novel_ClusteredSites), random clustered sites (Random_ClusteredSites), all motif sites in promoters (Novel_AllSites_Promoter), random control sites in promoters (Random_AllSites_Promoter), clustered motif sites in promoters (Novel_ClusteredSites_Promoter), and random clustered sites in promoters (Random_ClusteredSites_Promoter). For each motif site category and cell line, the average DHS across all motif sites is shown. (**a**) Heatmap of DHS of different motif site categories in all cell lines. (**b**) Zoomed-in plot for 10 cell lines from the bottom of (**a**).

DNase I hypersensitivity (DHS) is a hallmark for active cis-regulatory element^16^. We analyzed human DNase-seq data from the ENCODE project^10^ and asked whether the motif sites were associated with elevated level of DHS. The analysis involved a total of 193 cell lines. After normalization, DNase-seq signals from each cell line were extracted for the motif sites, random control sites, motif sites in promoters, and random control sites in promoters (Methods). Similarly, we also extracted DNase-seq signals for clustered motif sites, random clustered sites, clustered motif sites in promoters, and random clustered sites in promoters. For each cell line, the average DHS across all loci in each motif site category was computed and shown in Fig. 5. The figure shows that for all 193 cell lines, DHS at motif sites was higher than DHS at the random control sites. DHS at clustered motif sites was higher than DHS at the general motif sites as well as DHS at the clustered random control sites. It was also higher than or comparable to the DHS typically observed in promoter regions (i.e., DHS at random genomic sites in promoters and at random clustered sites in promoters). DHS at motif sites in promoters was even higher, and it was higher than the DHS at random genomic sites in promoters. Clustered motif sites in promoters were associated with the highest DHS. On average, DHS at the clustered motif sites in promoters was 3.75 times as high as the DHS at random clustered sites, and 1.61 times as high as the DHS at random clustered sites in promoter regions. We performed two-sample t-test in each cell line to test the hypothesis that DHS at the clustered motif sites within promoters is equal as opposed to higher compared to DHS at the clustered random control sites. The one-sided p-values after Bonferroni correction (adjusting for 193 tests) were less than 1e^–13^ for all cell lines. Similarly, in all cell lines, the t-test p-values for comparing the clustered motif sites in promoters with the random clustered sites in promoters were also less than 1e^–13^ after Bonferroni correction.

**Figure 6:**
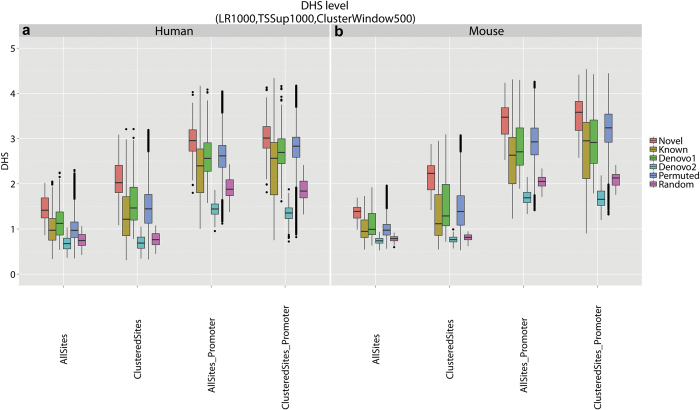
Comparisons of DNase I hypersensitivity between the new motif and control motifs. (**a**) DHS in 193 human ENCODE cell lines. (**b**) DHS in 53 mouse ENCODE cell lines. For each species, DHS were compared for four categories of motif sites: all motif sites (“AllSites”), clustered motif sites (“ClusteredSites”), all motif sites in promoters (“AllSites_Promoter”), and clustered motif sites in promoters (“ClusteredSites_Promoter”). Within each category, DHS was extracted for motif sites of the novel motif (“Novel”), eight known motifs (“Known”), three *de novo* class 1 motifs (“Denovo1”), three *de novo* class 2 motifs (“Denovo2”), 500 permuted motifs (“Permuted”), and random genomic controls (“Random”). For each motif, the average DHS across all motif sites was obtained. The distribution of this average DHS across all motifs and all cell lines in each motif category is shown using boxplot.

We further compared DHS of the new motif with DHS of the control motifs. For each motif category, Fig. 6 shows the distribution of the DHS level across the 193 ENCODE cell lines. Here DHS level of a motif in a cell line is summarized using the average DHS across all motif sites. Each boxplot in Fig. 6 consists of 193 × *K* data points where *K* is the number of motifs in the corresponding motif category (e.g., *K* = 500 for permuted motifs). The figure shows that the new motif on average had stronger DHS at its motif sites compared to different types of control motifs. In particular, DHS at the new motif was stronger than DHS at known motifs, which in turn was stronger than DHS at random genomic sites. Next, we defined the overall DHS level of a motif using its average DHS across all 193 cell lines. It turned out that only 0.8% of the 500 permuted motifs, 1 (SP1) of the 8 known motifs, 1 of the *de novo* class 1 motifs, and 0 of the *de novo* class 2 motifs had the same or higher level of overall DHS at their motif sites in promoters compared to the new motif. Similarly, only 1.2% of the 500 permuted motifs, 1 (SP1) of the 8 known motifs, 1 of the *de novo* class 1 motifs, and 0 of the *de novo* class 2 motifs had the same or higher level of overall DHS at their clustered motif sites in promoters compared to the new motif.

When the clustering tendency, phylogenetic conservation, promoter enrichment and DHS were considered jointly, the percentage of control motifs that were as extreme as or more extreme than the new motif with respect to all four features was even smaller. In fact, none of the permuted motifs, known motifs, and *de novo* class 2 motifs, and only one of the *de novo* class 1 motifs produced more motif site clusters (≥23,152) than the new motif, had the same or higher level of overall conservation and promoter enrichment at the clustered motif sites, and at the same time showed the same or higher level of overall DHS at their clustered motif sites in promoters compared to the new motif. The results for analyzing all motif sites were similar.

We repeated the same analyses on 53 ENCODE mouse cell lines^9^ and obtained essentially the same results (Fig. 6, Supplementary Figure S7). Based on the above analyses, we conclude that the clustered motif sites in promoters are very likely to be functional regulatory elements in the genome. Among the analyzed human cell lines, the global DHS level at clustered motif sites within promoters was the highest in neuroblastoma cell line SK-N-SH, olfactory neurosphere cell line, neuroblastoma cell line SK-N-SH (Retinoic Acid), colorectal adenocarcinoma (Caco-2) cell line, and hematopoietic progenitor cells (CD34+_Mobilized). Among the analyzed mouse cell lines, DHS level was very high in retina (C57BL/6 strain), E11.5 headless embryo (CD-1 strain), E0 undifferentiated mouse embryonic stem cells ZhBTc4 (129/Ola strain) and E11.5 mesoderm (CD-1 strain).

### Motif site clusters are associated with a large proportion of genes

Since motif site clusters found in promoter regions are highly unlikely to occur by chance and clustered motif sites are associated with high DHS, we label a protein-coding gene as a potential target of the motif if it has≥1 motif site cluster within its 1kb upstream TSS region. In this way, 7,205 genes (defined by unique Entrez identifiers) in human and 6,088 genes in mouse in the RefSeq database^17^ were annotated to be the targets of the motif, accounting for 33.8% and 32.0% of the protein-coding genes in the RefSeq database analyzed for each species. This large number of target genes suggests that the motif is likely to be a very common sequence element whose function is linked to a significant proportion of genes. Of note, among the 8 known transcription factor binding motifs, only SP1 was annotated with more target genes than the new motif by applying the same clustering and annotation criteria (Supplementary Table S4).

Gene ontology and gene set enrichment analysis performed using DAVID Bioinformatics Resources 6.7 tool^18^,^19^ (FDR ≤ 5%) identified a large number of functions enriched in the target genes of the motif including, for instance, cell morphogenesis, axonogenesis, ureteric bud development, kidney development, and differentiation among many others (Supplementary Tables S5, S6). The broad spectrum of enriched functions is likely due to the large number of target genes. Because of the large number of positive DAVID results, it is not immediately clear whether there is a single function that is the most important function of the motif.

We also tried a less stringent way to define target genes by relaxing the motif site cluster requirement. In the relaxed definition, a gene is called as a motif target if the gene has at least one motif site (instead of one motif site cluster) in its 1kb upstream TSS region. Based on this definition, the number of potential target genes increased to 9,660 in human and 8,121 in mouse, representing even larger proportions of genes in the genome associated with the motif.

### Robustness of conclusions

Finally, we evaluated whether our conclusions were sensitive to different choices of parameters.

First, we changed the likelihood ratio cutoff for mapping motif sites of the new motif from LR ≥ 1000 to LR ≥ 500 and LR ≥ 2000. Control motifs were also remapped to yield the same number of motif sites as the new motif (except NRSF for which we used motif sites obtained at LR ≥ 100). Supplementary Figures S8 and S9 show that regardless of the motif mapping criteria, the new motif consistently yielded higher number of motif site clusters and clustered motif sites compared to most control motifs, similar to Table 1. Next, we changed the window size for defining motif site clusters from 500 bp to 250 bp and 1000 bp. Supplementary Figures S10 and S11 show that changing the window size did not change the conclusion regarding the clustering tendency of the new motif. Thus, the observed clustering tendency of the new motif is robust to different choices of parameters.

Similarly, we evaluated phylogenetic conservation by using different motif mapping cutoffs and cluster window sizes. Regardless of the parameter choices, the observed phylogenetic conservation patterns of the new motif remained qualitatively the same (Supplementary Figures S12, S13).

We then examined the promoter enrichment. Supplementary Figures S14 and S15 show results obtained by using different motif mapping cutoffs, and Supplementary Figures S16 and S17 show results obtained by using different cluster window sizes. In addition, we also changed the promoter definition from 1 kb upstream of TSS to 0.5 kb, 1.5 kb, and 2 kb upstream of TSS, and the corresponding analysis results are shown in Supplementary Figures S18 and S19. In all these analyses, the conclusion regarding the promoter enrichment remained essentially the same.

Similarly, we evaluated the DHS level by using different motif mapping cutoffs, cluster window sizes, or promoter definitions. Different choices of parameters did not change the conclusion regarding the increased DHS level of the new motif (Supplementary Figures S20–S22).

In order to see how the conclusion that the motif is associated with a large proportion of genes in the genome depends on the target gene definition, we changed the motif mapping cutoff, cluster window size, and promoter definition as before. In addition, we also tried to define motif site clusters by requiring a minimal of three motif sites within a 250 bp, 500 bp or 1000 bp window (Methods). All these analyses resulted in a large number of target genes (Supplementary Table S7). For instance, if one requires each target gene to contain at least one motif site cluster, the number of target genes of the new motif ranged from 3,116 to 8,651 (14.6% - 40.6% of all genes) for human, and ranged from 2,502 to 7,266 (13.2% - 38.2% of all genes) for mouse. If one only requires each target gene to contain a motif site, the target gene number was even higher (Supplementary Table S7).

Together, these analyses show that conclusions we obtained in this study are robust and they are not tied to a specific parameter setting.

## Discussion

In summary, our computational analyses show that the new motif is highly enriched in promoter regions and has strong clustering tendency. The clustered motif sites are associated with increased DNase I hypersensitivity and phylogenetic conservation. These are all typical characteristics of functional regulatory elements in the genome. Therefore, our data suggest that the motif is highly likely to be part of the regulatory vocabulary in the human and mouse genomes. Of note, our analysis of GLI3 ChIP-chip data has also identified three other unknown motifs (i.e., the *de novo* class 1 motifs) which were enriched ≥2 folds in GLI3 binding regions compared to matched genomic control regions. It is possible that some or all of these motifs could also be part of the genome’s regulatory vocabulary. However, they are not the focus of this article since we are less confident about their potential of being real functional motifs. Compared to the new motif studied in this article, motifs 1 and 3 in the *de novo* class 1 in Supplementary Figure S1 had lower level of clustering tendency, conservation, promoter enrichment, and DHS. Motif 2 in the *de novo* class 1 indeed had the same or higher level of clustering tendency, conservation, promoter enrichment and DHS compared to the new motif. However, that motif had relatively low sequence specificity. Its total information content and per-nucleotide information content were only above 10.3% and 7.1% of the known motifs in TRNASFAC. This leads to some ambiguities regarding whether motif 2 is a high-quality real motif or a sequence pattern mixed with high-level of noise reported by the Gibbs motif sampler. In addition, the core sequence pattern GCGCACNYG of motif 2 shares some similarities with a segment (positions 9-17) of the new motif, suggesting that it might be a variant of the new motif.

The fact that motif site clusters of the new motif are very unlikely to occur by chance in promoters and a large number of the protein-coding genes are associated with motif site clusters indicate that the motif may have a function associated with a significant proportion of genes, highlighting its potential importance. For this reason, even though experimental investigation of the motif is beyond our capacity as a computational group, we believe that it is worthwhile to document this motif and hope that experimental biologists who are interested in this motif can further elucidate its exact function in the future.

## Methods

### *De novo* motif discovery

We extracted DNA sequences from the mouse genome for the GLI3 binding regions listed in Supplementary Table S1 and used the Gibbs motif sampler provided by CisGenome^15^ to search for enriched sequence motifs. With the motifs obtained, the relative enrichment level of each motif in GLI3 binding regions compared to matched negative control regions was computed using the method described in Ji et al^20^. Briefly, the negative control regions were chosen such that the distance between the control regions and their closest TSSs and the distance between the GLI3 binding sites and their closest TSSs had the same distribution. For each motif, CisGenome counted the number of motif sites in GLI3 binding regions (*n*_1_), the number of motif sites in matched control regions (*n*_2_), the total length of GLI3 binding regions after masking repeats by RepeatMasker (www.repeatmasker.org) (*L*_1_), and the total non-repeat length of matched control regions (*L*_2_). The number of motif sites per base pair was computed for both the GLI3 binding regions (*n*_1_/*L*_1_) and control regions (*n*_2_/*L*_2_) to characterize its occurrence rates. The ratio between the two occurrence rates (*r* = [*n*_1_*L*_2_]/[*n*_2_*L*_1_]) was calculated to represent the relative enrichment level of the motif. Motifs with relative enrichment level *r*  ≥ 2 were reported by CisGenome. These include the new motif, two known motifs GLI and SP1, and three *de novo* class 1 motifs shown in Supplementary Figure S1. The relative enrichment level *r*  ≥ 2 can be translated into FDR ≤ 50% for the discovered motif sites, that is, among every two motif sites for a motif with *r* ≥ 2, less than one is expected to occur by chance based on matched genomic controls. *r* < 2 corresponds to FDR > 50% (i.e., the majority of the reported motif sites are noise). This is the reason why *r*  ≥ 2 was chosen as the cutoff. To provide a control for our data analyses, we also relaxed the enrichment cutoff to *r*  ≥ 1.2 and obtained th*r*ee other motifs, listed as *de novo* class 2 motifs in Supplementary Figure S1.

To determine whether the motif is new, we used TOMTOM motif comparison tool^21^ to check if there was any known motif in the TRANSFAC, JASPAR and UniPROBE database that matched to the novel motif. TOMTOM was run using three different motif similarity measures including Pearson correlation coefficient, Euclidean distance and Sandelin-Wasserman similarity. The significance cutoff was set to E-value<10. This analysis did not find any motif with the same pattern as the new motif. Similarly, we also checked if the novel motif matched to the collection of 233 matrix profiles reported by Xie et al. which were derived by searching overrepresented motifs from human conserved non-coding elements^4^. In all these analyses, no existing motif was found to match the novel motif. Finally, we also visually examined sequence logos (both forward strands and reverse complement strands) of all motifs in TRNASFAC and JASPAR and did not find any motif whose logo resembles the logo of the new motif.

### Motif information content

The sequence specificity of a motif was characterized by its information content, including total information content and per-nucleotide information content. The total information content was defined as:


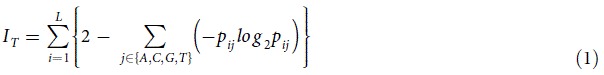


Here *L* is the length of the motif. Positions in the motif are indexed by *i*, nucleotide type is indexed by *j*, and *p*_*ij*_ represents the probability of observing nucleotide *j* at position *i*. The total information content is the sum of information contents of all positions, whereas the information content for each position is equal to two minus its Shannon entropy. If the A, C, G, and T occur with equal probability (1/4) at a position, the information content of that position is 0. If one nucleotide occurs with probability 1 and the other three occur with probability 0, the information content at the position achieves its maximal value which is 2.

The per-nucleotide information content was defined as:


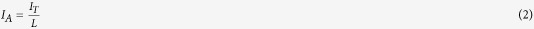


### Mapping motif to the genome and motif site clustering

Using CisGenome, the position-specific probability matrix in Supplementary Table S2 was mapped to mouse genome (mm9) and human genome (hg19). CisGenome uses an *L* bp sliding window to scan the genome (*L* is the motif length). At each genomic position, the probability of obtaining the observed DNA sequence from the motif model and the probability of generating the same sequence from a third-order Markov chain background model are computed. The ratio between these two probabilities gives the likelihood ratio (LR) between the motif model and the background model. All genomic sites with LR passing a user-specified cutoff are reported as motif sites. For our default analysis, the new motif was mapped using LR ≥ 1000 as the cutoff. The other motifs (except for NRSF) were mapped by adjusting the LR cutoff so that they produced the same number of motif sites as the new motif in order to meaningfully perform subsequent comparisons such as the number of motif site clusters. For NRSF, we only obtained 18,974 motif sites in human and 19,034 motif sites in mouse at the minimal likelihood ratio cutoff recommended by CisGenome (LR ≥ 100), and therefore we used LR ≥ 100 as the cutoff.

For each motif, if two motif sites overlapped, we only retained one of them (the one with higher likelihood ratio) to avoid redundancy. All analyses and numbers reported in this article were based on these non-redundant sets of motif sites. In our default analyses, motif site clusters were obtained by merging non-redundant motif sites separated by ≤500 bp.

### Control genomic sites and control motifs

To help interpret the observed characteristics of the new motif, we constructed two types of controls.

First, for each species, we generated a list of random genomic sites (denoted as “random sites”). These were obtained by randomly choosing the same number of genomic sites as the new motif from the non-repeat regions in the genome. Clusters of these random sites were obtained by applying the same criteria for creating clusters of the new motif sites. This resulted in “random clusters”. Sites in random clusters were called “random clustered sites”.

Second, we compiled a list of control motifs including 8 known motifs, 3 *de novo* class 1 motifs and 3 *de novo* class 2 motifs in Supplementary Figures S1 and S3. As described in Xie et al., another type of control can be created by permuting the probability matrix of the new motif 4. We generated 500 such control motifs by permuting the probability vectors from different positions of the new motif. All control motifs were analyzed in the same way as the new motif. They were mapped to the genome to produce the same number of motif sites as the new motif (except NRSF). The mapped motif sites were then used to produce motif site clusters using the clustering procedure described before.

### Analysis of phylogenetic conservation

Phylogenetic conservation was measured using phastCons scores downloaded from the UCSC genome browser^22^. The phastCons scores for human were based on multiple alignments between human genome and genomes from placental mammals. The phastCons scores for mouse were based on multiple alignments between mouse and 30 vertebrate genomes. The conservation score was linearly scaled to the interval [0, 255] so that the score for each position can be stored in one byte. The score 255 corresponds to the most conserved state. To generate Fig. 3a, for each position within and flanking the motif, the average conservation score across all motif sites were computed using CisGenome. Similarly, we characterized conservation for random control sites. To generate Fig. 3b, we summarized the overall conservation of each motif using the average conservation score of all its motif sites (or clustered motif sites), where the conservation score of a motif site is computed as the average phastCons score across all positions within the motif site. The overall conservation was computed for all control motifs. The distributions of the overall conservation for motifs in different motif categories were plotted in Fig. 3b.

### Analysis of motif locations

Genomic distributions of motif sites and motif site clusters (e.g., locations relative to TSS) were determined using CisGenome. With the coordinates of motif sites and clusters, CisGenome counted how many of them were located within 1kb upstream of transcription start site (TSSup1k), 1 kb downstream of transcription end site (TESdown1k), intragenic regions (regions between the transcription start and end sites of a gene), intergenic regions (regions other than intragenic regions). Here genes were defined based on RefSeq database downloaded from UCSC genome browser^22^.

### Analysis of DNase I hypersensitivity

DNase-seq data for 193 human cell lines and 53 mouse cell lines were downloaded from ENCODE. DHS for a genomic window is represented using the read count within the window. This read count was normalized by the total number of reads of each sample and then multiplied with a constant equal to the median total read count of all samples. The normalized read count *y* was log transformed after adding 1 (i.e., log_2_(*y* + 1)). Then the transformed counts from replicate samples were averaged to represent the DHS level for the window in a particular cell line.

In order to generate Fig. 5 and Supplementary Figure S7, we extracted a 200 bp window centered at the motif for each motif site. The DHS signals within these windows were then computed. The figures show the average DHS signal across all motif sites, motif sites in promoters, clustered motif sites, and clustered motif sites in promoters. We also computed the average DHS of random genomic sites, random sites in promoters, random clustered sites, and random clustered sites in promoters. These control regions were 200 bp windows obtained using the same approach as described above in the “control genomic sites and control motifs”. DHS levels for these control regions were compared with DHS levels of the motif sites.

In order to generate Fig. 6, we further computed the mean DHS level across all motif sites and across all the clustered motif sites for each control motif in the 193 ENCODE cell lines. We then plotted the distribution of the mean DHS level for different types of control motifs.

### Analysis of Robustness

We changed values of different parameters including the likelihood ratio cutoff for mapping the new motif, definition of motif site clusters, and definition of promoters. When one parameter was changed, the other parameters were hold as constant using their default values (LR ≥ 1000; cluster window size=500 bp; promoter = 1kb upstream of TSS). Analyses of random genomic sites and control motifs were also updated by matching the site number with the new motif and applying the new cluster and promoter definitions. All comparisons were then performed in the same way as described above.

## Author Contributions

H.J. and S.Z. conceived the study. S.Z. performed the analysis. F.D. processed the DNase-seq data. S.Z. and H.J. wrote the manuscript. All authors reviewed the manuscript.

## Additional Information

**How to cite this article**: Zhang, S. *et al.* A novel DNA sequence motif in human and mouse genomes. *Sci. Rep.* **5**, 10444; doi: 10.1038/srep10444 (2015).

## Supplementary Material

Supplementary Information

## Figures and Tables

**Figure 1:**
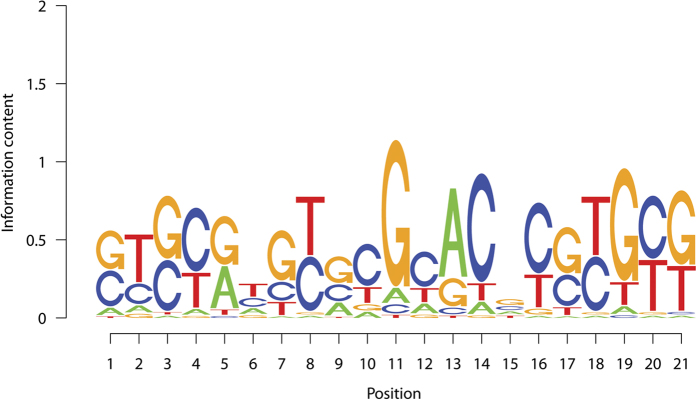
Sequence logo of the novel motif

**Table 1:** Summary of the number of motif sites, motif site clusters, and clustered motif sites and their percentage in promoter regions.

	**Motif sites**		**Clusters**		**Clustered Sites**	
	**TSSup1k**	**Total No.**	**TSSup1k**	**Total No.**	**TSSup1k**	**Total No.**
**Human (hg19)**						
New motif	11.28%	205283	15.71%	23152	22.44%	89915
Random Control	1.24%	205283	1.51%	9846	1.53%	20223
GLI	2.92%	205283	6.32%	14998	7.34%	34053
CTCF	5.70%	205283	11.42%	19047	15.07%	50267
NRSF	4.16%	18974	12.42%	451	11.57%	1003
FOXA1	0.87%	205283	0.83%	11271	0.81%	23660
MYC	2.48%	205283	4.56%	14524	4.81%	32778
ER	2.44%	205283	5.17%	14038	5.91%	31306
Oct4-Sox2	0.75%	205283	0.75%	12344	0.77%	25822
SP1	12.66%	205283	17.98%	23368	26.71%	83743
De novo class 1	7.22% (5.92%)	205283 (0)	10.83% (5.61%)	18840 (4643)	15.08% (8.55%)	61967 (39919)
De novo class 2	0.93% (0.10%)	205283 (0)	0.99% (0.10%)	13165 (1819)	1.01% (0.08%)	28007 (4323)
Permuted Motifs	4.61% (2.48%)	205283 (0)	9.65% (3.86%)	15047 (3018)	12.96% (5.98%)	40854 (16824)
**Mouse (mm9)**						
New motif	7.99%	212847	12.96%	21537	20.03%	72962
Random Control	1.11%	212847	1.32%	10719	1.37%	22061
GLI	2.17%	212847	4.31%	14721	5.17%	32447
CTCF	4.30%	212847	9.64%	18422	12.67%	44868
NRSF	3.32%	19034	9.51%	347	8.01%	824
FOXA1	0.85%	212847	0.75%	11796	0.75%	24647
MYC	1.83%	212847	3.19%	14442	3.40%	31320
ER	1.78%	212847	3.15%	14060	3.60%	30528
Oct4-Sox2	0.82%	212847	0.87%	12959	0.87%	27007
SP1	9.93%	212847	16.90%	22502	25.61%	69252
De novo class 1	5.17% (4.36%)	212847 (0)	8.47% (5.20%)	17881 (4266)	11.98% (9.09%)	52416 (31458)
De novo class 2	1.02% (0.15%)	212847 (0)	1.19% (0.47%)	13621 (1700)	1.20% (0.47%)	28605 (3877)
Permuted Motifs	3.4% (1.76%)	212847 (0)	7.23% (3.28%)	15155 (2756)	9.75% (5.27%)	37507 (13072)

Note: For *de novo* class 1, *de novo* class 2, and permuted motifs, the numbers shown are mean and standard deviation of all motifs in each category. The numbers in parentheses are the standard deviations.

## References

[b1] Matys V. *et al.* TRANSFAC: transcriptional regulation, from patterns to profiles. Nucleic Acids Research 31, 374–378 (2003). 12520026 10.1093/nar/gkg108 PMC165555

[b2] Hume M. A., Barrera L. A., Gisselbrecht S. S. & Bulyk M. L. UniPROBE, update 2015: new tools and content for the online database of protein-binding microarray data on protein–DNA interactions. Nucleic Acids Research (2014). 10.1093/nar/gku1045 PMC4383892 25378322

[b3] Mathelier A. *et al.* JASPAR 2014: an extensively expanded and updated open-access database of transcription factor binding profiles. Nucleic Acids Research 42, D142–D147 (2014 ). 24194598 10.1093/nar/gkt997 PMC3965086

[b4] Xie X. *et al.* Systematic discovery of regulatory motifs in conserved regions of the human genome, including thousands of CTCF insulator sites. Proc. Natl. Acad. Sci. U.S.A. 104, 7145–7150 (2007). 17442748 10.1073/pnas.0701811104 PMC1852749

[b5] Wang J. *et al.* Factorbook.org: a Wiki-based database for transcription factor-binding data generated by the ENCODE consortium. Nucleic Acids Research 41, D171–D176 (2013). 23203885 10.1093/nar/gks1221 PMC3531197

[b6] Berger M. F. *et al.* Compact, universal DNA microarrays to comprehensively determine transcription-factor binding site specificities. Nat Biotechnol 24, 1429–1435 (2006). 16998473 10.1038/nbt1246 PMC4419707

[b7] Hu S. *et al.* Profiling the Human Protein-DNA Interactome Reveals ERK2 as a Transcriptional Repressor of Interferon Signaling. Cell 139, 610–622 (2009). 19879846 10.1016/j.cell.2009.08.037 PMC2774939

[b8] Ji H. & Wong W. H. Computational Biology: Toward Deciphering Gene Regulatory Information in Mammalian Genomes. Biometrics 62, 645–663 (2006). 16984301 10.1111/j.1541-0420.2006.00625.x

[b9] Mouse ENCODE Consortium *et al.* An encyclopedia of mouse DNA elements (Mouse ENCODE). Genome Biol. 13, 418 (2012). 22889292 10.1186/gb-2012-13-8-418 PMC3491367

[b10] ENCODE Project Consortium. An integrated encyclopedia of DNA elements in the human genome. Nature 489, 57–74 (2012). 22955616 10.1038/nature11247 PMC3439153

[b11] Vokes S. A., Ji H., Wong W. H. & McMahon A. P. A genome-scale analysis of the cis-regulatory circuitry underlying sonic hedgehog-mediated patterning of the mammalian limb. Genes & Development 22, 2651–2663 (2008). 18832070 10.1101/gad.1693008 PMC2559910

[b12] Siepel A. *et al.* Evolutionarily conserved elements in vertebrate, insect, worm, and yeast genomes. Genome Research 15, 1034–1050 (2005). 16024819 10.1101/gr.3715005 PMC1182216

[b13] Carroll J. S. *et al.* Genome-wide analysis of estrogen receptor binding sites. Nat Genet 38, 1289–1297 (2006). 17013392 10.1038/ng1901

[b14] Eeckhoute J. *et al.* Cell-type selective chromatin remodeling defines the active subset of FOXA1-bound enhancers. Genome Research 19, 372–380 (2009). 19129543 10.1101/gr.084582.108 PMC2661808

[b15] Ji H. *et al.* An integrated software system for analyzing ChIP-chip and ChIP-seq data. Nat Biotechnol 26, 1293–1300 (2008). 18978777 10.1038/nbt.1505 PMC2596672

[b16] Song L. & Crawford G. E. DNase-seq: a high-resolution technique for mapping active gene regulatory elements across the genome from mammalian cells. Cold Spring Harbor Protocols 2010, pdb.prot5384 (2010). 20150147 10.1101/pdb.prot5384 PMC3627383

[b17] Pruitt K. D. *et al.* RefSeq: an update on mammalian reference sequences. Nucleic Acids Research 42, D756–D763 (2014). 24259432 10.1093/nar/gkt1114 PMC3965018

[b18] Huang D. W., Sherman B. T. & Lempicki R. A. Systematic and integrative analysis of large gene lists using DAVID bioinformatics resources. Nat Protoc 4, 44–57 (2008). 10.1038/nprot.2008.211 19131956

[b19] Huang D. W., Sherman B. T. & Lempicki R. A. Bioinformatics enrichment tools: paths toward the comprehensive functional analysis of large gene lists. Nucleic Acids Research 37, 1–13 (2009). 19033363 10.1093/nar/gkn923 PMC2615629

[b20] Ji H., Vokes S. A. & Wong W. H. A comparative analysis of genome-wide chromatin immunoprecipitation data for mammalian transcription factors. Nucleic Acids Research 34, e146–e146 (2006). 17090591 10.1093/nar/gkl803 PMC1669715

[b21] Gupta S., Stamatoyannopoulos J. A., Bailey T. L. & Noble W. Quantifying similarity between motifs. Genome Biol. 8, R24 (2007). 17324271 10.1186/gb-2007-8-2-r24 PMC1852410

[b22] Kent W. J. *et al.* The human genome browser at UCSC. Genome Research 12, 996–1006 (2002). 12045153 10.1101/gr.229102 PMC186604

