# Supplementary material for: A novel DNA sequence motif in human and mouse genomes
Source: Sci Rep. 2015 May 20;5:10444. doi: 10.1038/srep10444 (PMC4438489; doi:10.1038/srep10444)
Supplement: Supplementary Information [file srep10444-s1.pdf]

A novel DNA sequence motif in human and mouse genomes
Supplementary Information

Shilu Zhang, Fang Du, Hongkai Ji

This file contains Supplementary Tables S1-S7 and Supplementary Figures S1-S22.

Supplementary Table S1. List of the GLI3 binding regions in mouse genome used for motif discovery.

Chromosome	Start	End
chr1	11967543	11968294
chr1	12681804	12682737
chr1	12682959	12683991
chr1	99947577	99948123
chr1	158870096	158871587
chr1	158894092	158895134
chr1	158978121	158979128
chr2	49865906	49866397
chr2	49922054	49922998
chr2	74510348	74513248
chr2	74521081	74522818
chr2	74523209	74524151
chr2	113480728	113481771
chr2	132966468	132967230
chr2	133460976	133461956
chr2	136913320	136914493
chr2	136943053	136944349
chr2	137073620	137074592
chr2	137647050	137647640
chr3	31090209	31091135
chr3	83575921	83576564
chr3	89095103	89095614
chr4	41607435	41608775
chr4	58192749	58193298
chr4	63100725	63101896
chr4	63120025	63121222
chr4	116762842	116763387
chr4	116767756	116769455
chr4	116770624	116771673
chr4	134702643	134703398
chr4	134722198	134723676
chr5	3341406	3344432
chr5	75550461	75550850
chr5	93157314	93157906
chr5	99666396	99667520
chr5	99806901	99807930
chr5	100019846	100020653
chr5	100147162	100147974
chr5	100158140	100159225
chr5	119459440	119459975
chr5	119638205	119638938

Chromosome	Start	End
chr5	119710986	119711681
chr5	119829896	119830855
chr5	119872769	119873133
chr5	119874440	119875363
chr5	119922290	119922879
chr5	119930009	119931952
chr5	119984875	119985137
chr5	120120647	120121700
chr6	65621997	65623607
chr6	85441401	85442227
chr7	100241948	100242468
chr7	105963790	105964466
chr7	105963829	105964495
chr7	144489180	144490349
chr8	59035169	59035757
chr8	59492675	59493249
chr8	59612605	59613489
chr8	59806684	59807535
chr8	59811888	59814571
chr8	59821100	59822516
chr8	59883775	59884720
chr8	82505892	82506861
chr8	82579645	82580709
chr8	82837835	82839819
chr8	82841604	82843044
chr8	87178799	87180894
chr8	87184300	87185670
chr8	87188798	87189773
chr8	87266138	87268103
chr8	91530129	91530773
chr8	91907152	91908456
chr8	92021858	92022521
chr8	92156002	92156569
chr8	92164810	92166008
chr8	92201008	92202017
chr8	92207209	92207948
chr9	21651091	21652237
chr9	62704861	62706723
chr10	8167283	8167944
chr10	8170047	8170765
chr10	8533579	8534596
chr10	29506045	29506937
chr10	31165049	31165879

Chromosome	Start	End
chr10	44128644	44129545
chr10	126775842	126779541
chr11	51185041	51185913
chr11	85244305	85244913
chr11	85445883	85447203
chr11	85526140	85527468
chr11	85547284	85549041
chr11	85671205	85671784
chr11	87961917	87963166
chr11	87969952	87970601
chr11	87974641	87975780
chr11	87993458	87994563
chr11	88550748	88552213
chr11	88554983	88556007
chr12	9374602	9376517
chr12	9437327	9438298
chr12	9581582	9583469
chr12	9585346	9587478
chr12	9628615	9629320
chr12	10054828	10055516
chr12	10098243	10098457
chr12	74467950	74469864
chr12	74554252	74554968
chr12	74612412	74613189
chr12	74622006	74624019
chr12	82110469	82111459
chr12	110153073	110153711
chr13	29865531	29866136
chr13	63625844	63627686
chr13	63629202	63630949
chr13	63659332	63660350
chr13	63663502	63666653
chr13	63667337	63669216
chr13	63669469	63671820
chr13	63672903	63676180
chr13	63820647	63821534
chr13	63826537	63827608
chr13	64002197	64003762
chr13	64052129	64054058
chr13	64070804	64071736
chr13	64081424	64082322
chr13	64121775	64122612
chr14	27880574	27881825

Chromosome	Start	End
chr14	27882530	27883140
chr15	35222856	35224633
chr15	83496075	83498149
chr15	83530313	83531054
chr15	83552526	83553160
chr17	23832079	23833404
chr17	75288326	75289229
chr17	75404106	75404967
chr18	58819407	58820044
chr18	58996351	58997242
chr18	81287650	81288419
chr18	84932994	84934834
chr18	84938210	84938695
chr19	23207617	23208592

Supplementary Table S2. Position-specific probability matrix of the novel motif

Position	**A**	**C**	**G**	**T**
1	0.086	0.431	0.466	0.017
2	0.086	0.259	0.052	0.603
3	0.017	0.431	0.534	0.017
4	0.052	0.603	0.017	0.328
5	0.431	0.017	0.500	0.052
6	0.190	0.293	0.086	0.431
7	0.017	0.224	0.603	0.155
8	0.017	0.466	0.017	0.500
9	0.224	0.293	0.466	0.017
10	0.086	0.603	0.086	0.224
11	0.086	0.052	0.845	0.017
12	0.155	0.569	0.052	0.224
13	0.707	0.052	0.224	0.017
14	0.086	0.776	0.017	0.121
15	0.190	0.259	0.397	0.155
16	0.017	0.638	0.052	0.293
17	0.017	0.362	0.534	0.086
18	0.017	0.466	0.017	0.500
19	0.052	0.017	0.776	0.155
20	0.017	0.534	0.017	0.431
21	0.017	0.017	0.603	0.362

Supplementary Table S3. Physical distribution of the novel motif in human and mouse genomes

	Intergenic	Intragenic	TSSup1k	TESdown1k	Total No.
Human					
All sites	48.23%	51.77%	11.28%	1.25%	205283
Random sites	54.41%	45.59%	1.24%	1.08%	205283
Clusters	43.28%	56.72%	15.71%	1.37%	23152
Random clusters	53.98%	46.02%	1.51%	1.22%	9846
Clustered sites	43.04%	56.96%	22.44%	1.14%	89915
Random clustered sites	54.01%	45.99%	1.53%	1.25%	20223
Mouse					
All sites	52.57%	47.43%	7.99%	1.11%	212847
Random sites	60.77%	39.23%	1.11%	0.98%	212847
Clusters	45.77%	54.23%	12.96%	1.37%	21537
Random clusters	59.02%	40.98%	1.32%	1.05%	10719
Clustered sites	46.60%	53.40%	20.03%	1.08%	72962
Random clustered sites	59.01%	40.99%	1.37%	1.01%	22061

Supplementary Table S4. Number of annotated target genes for the new motif and known motifs.

Motif name	Gene No. (Allsites)[1]	Geno No. (Clusters) [2]
Human hg19		
Novel motif	9660	7205
GLI	4556	1354
CTCF	7165	3545
NRSF	730	70
FOXA1	1657	110
MYC	4138	861
ER	3993	1039
Oct4Sox2	1413	108
SP1	10649	7687
Mouse mm9		
Novel motif	8121	6088
GLI	3790	1564
CTCF	6151	3856
NRSF	600	73
FOXA1	1683	197
MYC	3286	982
ER	3225	1122
Oct4Sox2	1600	239
SP1	9610	7567

Note:
1. Gene No. (All sites) corresponds to the number of target genes annotated using all motif sites.
2. Gene No. (Clusters) corresponds to the number of target genes annotated using all motif site clusters.

Supplementary Table S5. DAVID functional enrichment analysis using human target genes of the new motif annotated based on motif site clusters.

Category	Term	Fold Enrichment[1]	FDR[2]
**SP_PIR_KEYWORDS**	phosphoprotein	1.27	1.34E-29
**SP_PIR_KEYWORDS**	acetylation	1.31	3.82E-09
**UP_SEQ_FEATURE**	splice variant	1.14	1.02E-07
**SP_PIR_KEYWORDS**	alternative splicing	1.14	1.34E-07
**SP_PIR_KEYWORDS**	nucleus	1.20	1.81E-06
**GOTERM_BP_FAT**	GO:0048812~neuron projection morphogenesis	2.03	2.87E-05
**GOTERM_CC_FAT**	GO:0070013~intracellular organelle lumen	1.30	4.70E-05
**GOTERM_BP_FAT**	GO:0048858~cell projection morphogenesis	1.93	6.02E-05
**GOTERM_CC_FAT**	GO:0031981~nuclear lumen	1.34	8.66E-05
**GOTERM_CC_FAT**	GO:0043233~organelle lumen	1.29	1.22E-04
**GOTERM_BP_FAT**	GO:0007409~axonogenesis	2.03	1.33E-04
**GOTERM_CC_FAT**	GO:0031974~membrane-enclosed lumen	1.28	2.47E-04
**GOTERM_BP_FAT**	GO:0032990~cell part morphogenesis	1.85	3.97E-04
**GOTERM_BP_FAT**	GO:0048667~cell morphogenesis involved in neuron differentiation	1.94	4.76E-04
**GOTERM_BP_FAT**	GO:0030030~cell projection organization	1.68	5.14E-04
**GOTERM_BP_FAT**	GO:0048666~neuron development	1.71	6.42E-04
**GOTERM_BP_FAT**	GO:0031175~neuron projection development	1.82	8.69E-04
**SP_PIR_KEYWORDS**	differentiation	1.59	1.14E-03
**GOTERM_BP_FAT**	GO:0030182~neuron differentiation	1.60	1.28E-03
**GOTERM_CC_FAT**	GO:0005654~nucleoplasm	1.40	2.13E-03
**SP_PIR_KEYWORDS**	developmental protein	1.41	3.95E-03
**GOTERM_BP_FAT**	GO:0001657~ureteric bud development	3.42	5.31E-03
**SP_PIR_KEYWORDS**	Transcription	1.23	5.73E-03
**GOTERM_BP_FAT**	GO:0000904~cell morphogenesis involved in differentiation	1.77	6.11E-03
**UP_SEQ_FEATURE**	mutagenesis site	1.23	8.39E-03
**GOTERM_BP_FAT**	GO:0007411~axon guidance	2.18	0.02
**GOTERM_BP_FAT**	GO:0000902~cell morphogenesis	1.59	0.02
**SP_PIR_KEYWORDS**	transcription regulation	1.22	0.02
**GOTERM_BP_FAT**	GO:0001822~kidney development	2.22	0.03
**SP_PIR_KEYWORDS**	cytoplasm	1.16	0.03
**GOTERM_BP_FAT**	GO:0001655~urogenital system development	2.12	0.03
**SP_PIR_KEYWORDS**	neurogenesis	1.94	0.04
**GOTERM_CC_FAT**	GO:0044451~nucleoplasm part	1.45	0.04
**UP_SEQ_FEATURE**	compositionally biased region:Ser-rich	1.52	0.04
**GOTERM_BP_FAT**	GO:0001656~metanephros development	2.87	0.05
**GOTERM_MF_FAT**	GO:0003700~transcription factor activity	1.31	0.06
**SP_PIR_KEYWORDS**	golgi apparatus	1.41	0.07
**GOTERM_BP_FAT**	GO:0006357~regulation of transcription from	1.36	0.08

Category	Term	Fold Enrichment[1]	FDR[2]
	RNA polymerase II promoter		
**GOTERM_CC_FAT**	GO:0030424~axon	1.87	0.08
**UP_SEQ_FEATURE**	domain:RRM 1	2.13	0.08
**UP_SEQ_FEATURE**	domain:RRM 2	2.13	0.08
**OMIM_DISEASE**	Genome-wide association analysis of susceptibility and clinical phenotype in multiple sclerosis	3.12	0.09
**GOTERM_BP_FAT**	GO:0032989~cellular component morphogenesis	1.51	0.09
**GOTERM_MF_FAT**	GO:0030528~transcription regulator activity	1.23	0.09
**GOTERM_MF_FAT**	GO:0003677~DNA binding	1.18	0.10
**SP_PIR_KEYWORDS**	transferase	1.25	0.10
**GOTERM_BP_FAT**	GO:0006350~transcription	1.19	0.11
**GOTERM_MF_FAT**	GO:0003723~RNA binding	1.35	0.14
**GOTERM_BP_FAT**	GO:0045449~regulation of transcription	1.16	0.14
**GOTERM_CC_FAT**	GO:0005794~Golgi apparatus	1.31	0.21
**GOTERM_CC_FAT**	GO:0012505~endomembrane system	1.33	0.22
**SP_PIR_KEYWORDS**	rna-binding	1.40	0.23
**UP_SEQ_FEATURE**	domain:RRM 3	2.55	0.35
**GOTERM_BP_FAT**	GO:0060284~regulation of cell development	1.66	0.49
**GOTERM_CC_FAT**	GO:0005667~transcription factor complex	1.65	0.54
**GOTERM_BP_FAT**	GO:0008104~protein localization	1.28	0.61
**OMIM_DISEASE**	Many sequence variants affecting diversity of adult human height	1.82	0.63
**GOTERM_BP_FAT**	GO:0050767~regulation of neurogenesis	1.72	0.65
**GOTERM_CC_FAT**	GO:0044454~nuclear chromosome part	1.87	0.66
**GOTERM_MF_FAT**	GO:0043565~sequence-specific DNA binding	1.34	0.66
**GOTERM_BP_FAT**	GO:0051960~regulation of nervous system development	1.66	0.68
**UP_SEQ_FEATURE**	compositionally biased region:Ala-rich	1.76	0.78
**GOTERM_BP_FAT**	GO:0045184~establishment of protein localization	1.30	0.80
**SP_PIR_KEYWORDS**	DNA binding	1.47	0.83
**UP_SEQ_FEATURE**	repeat:HEAT 2	2.32	0.95
**UP_SEQ_FEATURE**	repeat:HEAT 1	2.32	0.95
**UP_SEQ_FEATURE**	compositionally biased region:Poly-Ala	1.44	0.95
**GOTERM_BP_FAT**	GO:0046907~intracellular transport	1.32	1.14
**SP_PIR_KEYWORDS**	repressor	1.40	1.17
**GOTERM_BP_FAT**	GO:0000122~negative regulation of transcription from RNA polymerase II promoter	1.53	1.18
**GOTERM_BP_FAT**	GO:0019226~transmission of nerve impulse	1.44	1.35
**GOTERM_CC_FAT**	GO:0005829~cytosol	1.21	1.38
**GOTERM_MF_FAT**	GO:0048037~cofactor binding	1.53	1.48
**GOTERM_BP_FAT**	GO:0015031~protein transport	1.28	1.50
**GOTERM_BP_FAT**	GO:0035295~tube development	1.57	1.53
**GOTERM_CC_FAT**	GO:0044427~chromosomal part	1.42	1.57

Category	Term	Fold Enrichment[1]	FDR[2]
**GOTERM_BP_FAT**	GO:0010720~positive regulation of cell development	2.12	1.60
**GOTERM_CC_FAT**	GO:0043228~non-membrane-bounded organelle	1.14	1.66
**GOTERM_CC_FAT**	GO:0043232~intracellular non-membrane-bounded organelle	1.14	1.66
**SP_PIR_KEYWORDS**	kinase	1.30	1.77
**GOTERM_BP_FAT**	GO:0007268~synaptic transmission	1.47	1.84
**GOTERM_BP_FAT**	GO:0009954~proximal/distal pattern formation	3.18	1.85
**GOTERM_MF_FAT**	GO:0015631~tubulin binding	1.87	2.05
**GOTERM_CC_FAT**	GO:0000790~nuclear chromatin	2.26	2.08
**KEGG_PATHWAY**	hsa05200:Pathways in cancer	1.41	2.17
**SP_PIR_KEYWORDS**	wnt signaling pathway	1.77	2.18
**GOTERM_BP_FAT**	GO:0007389~pattern specification process	1.50	2.21
**SP_PIR_KEYWORDS**	zinc-finger	1.17	2.28
**GOTERM_BP_FAT**	GO:0048732~gland development	1.73	2.46
**GOTERM_BP_FAT**	GO:0006396~RNA processing	1.33	2.52
**GOTERM_BP_FAT**	GO:0006913~nucleocytoplasmic transport	1.66	2.58
**GOTERM_BP_FAT**	GO:0060562~epithelial tube morphogenesis	2.09	2.73
**GOTERM_BP_FAT**	GO:0002009~morphogenesis of an epithelium	1.84	2.92
**SP_PIR_KEYWORDS**	transcription factor	2.08	3.17
**GOTERM_BP_FAT**	GO:0007049~cell cycle	1.26	3.22
**GOTERM_MF_FAT**	GO:0000166~nucleotide binding	1.14	3.27
**GOTERM_BP_FAT**	GO:0051169~nuclear transport	1.64	3.30
**SP_PIR_KEYWORDS**	dna-binding	1.16	3.45
**GOTERM_BP_FAT**	GO:0050769~positive regulation of neurogenesis	2.14	3.51
**GOTERM_BP_FAT**	GO:0045934~negative regulation of nucleobase, nucleoside, nucleotide and nucleic acid metabolic process	1.33	3.61
**GOTERM_BP_FAT**	GO:0070727~cellular macromolecule localization	1.37	3.71
**GOTERM_BP_FAT**	GO:0016055~Wnt receptor signaling pathway	1.70	3.72
**GOTERM_BP_FAT**	GO:0006886~intracellular protein transport	1.39	3.72
**GOTERM_BP_FAT**	GO:0051172~negative regulation of nitrogen compound metabolic process	1.32	3.90
**GOTERM_BP_FAT**	GO:0051276~chromosome organization	1.33	4.11
**SP_PIR_KEYWORDS**	chromatin regulator	1.52	4.16
**GOTERM_MF_FAT**	GO:0008134~transcription factor binding	1.32	4.17
**BIOCARTA**	h_ionPathway:Ion Channel and Phorbal Esters Signaling Pathway	5.96	4.21
**GOTERM_BP_FAT**	GO:0016071~mRNA metabolic process	1.38	4.22
**UP_SEQ_FEATURE**	compositionally biased region:Gln-rich	1.65	4.47
**KEGG_PATHWAY**	hsa00240:Pyrimidine metabolism	1.78	4.55
**GOTERM_BP_FAT**	GO:0034613~cellular protein localization	1.36	4.59
**UP_SEQ_FEATURE**	domain:DEP	3.36	4.85
**GOTERM_BP_FAT**	GO:0021871~forebrain regionalization	4.23	4.86

Category	Term	Fold Enrichment[1]	FDR[2]
**INTERPRO**	IPR016040:NAD(P)-binding domain	1.63	4.88

Note:
1. Fold enrichment characterizes the ratio between the percentage of motif target genes that belong to the gene set in question and the percentage of all genes in the genome that belong to the gene set in question.
2. In the FDR column, 2 means FDR = 2%, and 0.2 means FDR = 0.2%.

Supplementary Table S6. DAVID functional enrichment analysis using mouse target genes of the new motif annotated based on motif site clusters.

Category	Term	Fold Enrichment[1]	FDR[2]
**SP_PIR_KEYWORDS**	phosphoprotein	1.39	1.36E-58
**SP_PIR_KEYWORDS**	nucleus	1.32	2.77E-18
**SP_PIR_KEYWORDS**	acetylation	1.44	4.33E-18
**GOTERM_MF_FAT**	GO:0000166~nucleotide binding	1.37	3.26E-11
**SP_PIR_KEYWORDS**	cytoplasm	1.30	8.63E-11
**GOTERM_MF_FAT**	GO:0030528~transcription regulator activity	1.48	5.31E-09
**SP_PIR_KEYWORDS**	alternative splicing	1.20	3.09E-08
**GOTERM_MF_FAT**	GO:0003677~DNA binding	1.35	1.36E-07
**SP_PIR_KEYWORDS**	transcription regulation	1.38	1.80E-07
**GOTERM_MF_FAT**	GO:0032553~ribonucleotide binding	1.34	2.59E-07
**GOTERM_MF_FAT**	GO:0032555~purine ribonucleotide binding	1.34	2.59E-07
**GOTERM_MF_FAT**	GO:0017076~purine nucleotide binding	1.33	3.65E-07
**SP_PIR_KEYWORDS**	ubl conjugation	1.68	4.64E-07
**GOTERM_CC_FAT**	GO:0043232~intracellular non-membrane-bounded organelle	1.32	4.65E-07
**GOTERM_CC_FAT**	GO:0043228~non-membrane-bounded organelle	1.32	4.65E-07
**GOTERM_BP_FAT**	GO:0010629~negative regulation of gene expression	1.79	4.66E-07
**SP_PIR_KEYWORDS**	nucleotide-binding	1.35	7.70E-07
**GOTERM_BP_FAT**	GO:0000902~cell morphogenesis	1.91	8.34E-07
**GOTERM_CC_FAT**	GO:0005739~mitochondrion	1.40	8.80E-07
**KEGG_PATHWAY**	mmu05200:Pathways in cancer	1.89	1.25E-06
**GOTERM_BP_FAT**	GO:0045934~negative regulation of nucleobase, nucleoside, nucleotide and nucleic acid metabolic process	1.77	2.70E-06
**GOTERM_BP_FAT**	GO:0009890~negative regulation of biosynthetic process	1.72	3.98E-06
**GOTERM_BP_FAT**	GO:0045449~regulation of transcription	1.28	4.45E-06
**GOTERM_BP_FAT**	GO:0031327~negative regulation of cellular biosynthetic process	1.72	4.59E-06
**GOTERM_BP_FAT**	GO:0051172~negative regulation of nitrogen compound metabolic process	1.75	4.95E-06
**GOTERM_BP_FAT**	GO:0010605~negative regulation of macromolecule metabolic process	1.65	5.26E-06
**GOTERM_CC_FAT**	GO:0031974~membrane-enclosed lumen	1.41	6.73E-06
**GOTERM_BP_FAT**	GO:0016481~negative regulation of transcription	1.76	1.08E-05
**GOTERM_CC_FAT**	GO:0043233~organelle lumen	1.40	2.02E-05
**GOTERM_CC_FAT**	GO:0070013~intracellular organelle lumen	1.40	2.50E-05
**GOTERM_BP_FAT**	GO:0000904~cell morphogenesis involved in differentiation	2.02	2.50E-05
**GOTERM_BP_FAT**	GO:0010558~negative regulation of macromolecule biosynthetic process	1.69	2.81E-05

Category	Term	Fold Enrichment[1]	FDR[2]
**GOTERM_CC_FAT**	GO:0005694~chromosome	1.74	3.17E-05
**GOTERM_BP_FAT**	GO:0032989~cellular component morphogenesis	1.76	3.80E-05
**GOTERM_CC_FAT**	GO:0005794~Golgi apparatus	1.52	3.97E-05
**GOTERM_BP_FAT**	GO:0030030~cell projection organization	1.79	5.16E-05
**GOTERM_BP_FAT**	GO:0016568~chromatin modification	1.92	7.60E-05
**SP_PIR_KEYWORDS**	zinc-finger	1.36	1.02E-04
**GOTERM_BP_FAT**	GO:0007242~intracellular signaling cascade	1.43	1.02E-04
**SP_PIR_KEYWORDS**	dna-binding	1.33	1.04E-04
**GOTERM_CC_FAT**	GO:0031981~nuclear lumen	1.44	1.18E-04
**GOTERM_CC_FAT**	GO:0012505~endomembrane system	1.58	1.19E-04
**GOTERM_BP_FAT**	GO:0006325~chromatin organization	1.77	1.25E-04
**GOTERM_BP_FAT**	GO:0006357~regulation of transcription from RNA polymerase II promoter	1.53	1.32E-04
**GOTERM_BP_FAT**	GO:0007049~cell cycle	1.53	1.36E-04
**GOTERM_CC_FAT**	GO:0005829~cytosol	1.56	1.69E-04
**GOTERM_BP_FAT**	GO:0008104~protein localization	1.46	1.86E-04
**GOTERM_CC_FAT**	GO:0015630~microtubule cytoskeleton	1.62	2.24E-04
**GOTERM_BP_FAT**	GO:0051276~chromosome organization	1.65	2.45E-04
**GOTERM_CC_FAT**	GO:0031090~organelle membrane	1.44	2.95E-04
**GOTERM_BP_FAT**	GO:0048858~cell projection morphogenesis	1.96	3.13E-04
**SP_PIR_KEYWORDS**	repressor	1.65	3.61E-04
**GOTERM_BP_FAT**	GO:0032990~cell part morphogenesis	1.93	3.62E-04
**GOTERM_BP_FAT**	GO:0007389~pattern specification process	1.78	4.18E-04
**GOTERM_BP_FAT**	GO:0009792~embryonic development ending in birth or egg hatching	1.62	5.08E-04
**GOTERM_CC_FAT**	GO:0044431~Golgi apparatus part	1.89	5.24E-04
**GOTERM_BP_FAT**	GO:0006350~transcription	1.27	5.78E-04
**GOTERM_BP_FAT**	GO:0006913~nucleocytoplasmic transport	2.43	6.14E-04
**SP_PIR_KEYWORDS**	golgi apparatus	1.52	6.22E-04
**KEGG_PATHWAY**	mmu04010:MAPK signaling pathway	1.80	7.09E-04
**GOTERM_BP_FAT**	GO:0000122~negative regulation of transcription from RNA polymerase II promoter	1.85	8.69E-04
**GOTERM_MF_FAT**	GO:0032561~guanyl ribonucleotide binding	1.67	9.09E-04
**GOTERM_MF_FAT**	GO:0019001~guanyl nucleotide binding	1.67	9.09E-04
**GOTERM_MF_FAT**	GO:0005525~GTP binding	1.67	1.07E-03
**GOTERM_BP_FAT**	GO:0051253~negative regulation of RNA metabolic process	1.72	1.09E-03
**GOTERM_BP_FAT**	GO:0043009~chordate embryonic development	1.60	1.09E-03
**GOTERM_BP_FAT**	GO:0051169~nuclear transport	2.38	1.09E-03
**GOTERM_CC_FAT**	GO:0031967~organelle envelope	1.53	1.15E-03
**GOTERM_CC_FAT**	GO:0044427~chromosomal part	1.71	1.30E-03
**GOTERM_BP_FAT**	GO:0048667~cell morphogenesis involved in	1.96	1.33E-03

Category	Term	Fold Enrichment[1]	FDR[2]
	neuron differentiation		
**GOTERM_BP_FAT**	GO:0048666~neuron development	1.73	1.39E-03
**GOTERM_CC_FAT**	GO:0031975~envelope	1.52	1.40E-03
**GOTERM_BP_FAT**	GO:0007264~small GTPase mediated signal transduction	1.79	1.43E-03
**SP_PIR_KEYWORDS**	Transcription	1.26	1.44E-03
**SP_PIR_KEYWORDS**	gtp-binding	1.69	1.52E-03
**SP_PIR_KEYWORDS**	cytoskeleton	1.49	1.58E-03
**GOTERM_BP_FAT**	GO:0045892~negative regulation of transcription, DNA-dependent	1.71	1.65E-03
**SP_PIR_KEYWORDS**	chromatin regulator	1.89	2.31E-03
**GOTERM_BP_FAT**	GO:0045184~establishment of protein localization	1.45	2.38E-03
**GOTERM_BP_FAT**	GO:0034504~protein localization in nucleus	2.77	2.41E-03
**GOTERM_BP_FAT**	GO:0048812~neuron projection morphogenesis	1.95	2.44E-03
**GOTERM_BP_FAT**	GO:0015031~protein transport	1.46	2.48E-03
**GOTERM_BP_FAT**	GO:0007409~axonogenesis	1.99	2.82E-03
**GOTERM_BP_FAT**	GO:0030182~neuron differentiation	1.59	3.12E-03
**GOTERM_BP_FAT**	GO:0060284~regulation of cell development	2.00	3.14E-03
**GOTERM_BP_FAT**	GO:0035295~tube development	1.75	3.55E-03
**GOTERM_BP_FAT**	GO:0001822~kidney development	2.24	3.89E-03
**GOTERM_MF_FAT**	GO:0003700~transcription factor activity	1.41	4.28E-03
**SP_PIR_KEYWORDS**	isopeptide bond	1.70	4.62E-03
**GOTERM_MF_FAT**	GO:0032559~adenyl ribonucleotide binding	1.28	4.74E-03
**GOTERM_BP_FAT**	GO:0031175~neuron projection development	1.82	5.23E-03
**GOTERM_MF_FAT**	GO:0030554~adenyl nucleotide binding	1.27	5.65E-03
**GOTERM_BP_FAT**	GO:0031110~regulation of microtubule polymerization or depolymerization	3.71	5.85E-03
**GOTERM_MF_FAT**	GO:0005524~ATP binding	1.28	6.97E-03
**GOTERM_BP_FAT**	GO:0033365~protein localization in organelle	2.27	7.76E-03
**GOTERM_CC_FAT**	GO:0005654~nucleoplasm	1.46	7.87E-03
**GOTERM_BP_FAT**	GO:0050767~regulation of neurogenesis	2.06	8.00E-03
**GOTERM_MF_FAT**	GO:0001883~purine nucleoside binding	1.26	8.46E-03
**GOTERM_MF_FAT**	GO:0008134~transcription factor binding	1.69	8.87E-03
**KEGG_PATHWAY**	mmu04310:Wnt signaling pathway	2.00	9.33E-03
**SP_PIR_KEYWORDS**	developmental protein	1.37	0.010
**GOTERM_BP_FAT**	GO:0051173~positive regulation of nitrogen compound metabolic process	1.48	0.010
**GOTERM_BP_FAT**	GO:0045935~positive regulation of nucleobase, nucleoside, nucleotide and nucleic acid metabolic process	1.49	0.010
**GOTERM_MF_FAT**	GO:0001882~nucleoside binding	1.26	0.010
**GOTERM_MF_FAT**	GO:0008270~zinc ion binding	1.22	0.010
**GOTERM_BP_FAT**	GO:0044265~cellular macromolecule	1.44	0.012

Category	Term	Fold Enrichment[1]	FDR[2]
	catabolic process		
**GOTERM_BP_FAT**	GO:0070507~regulation of microtubule cytoskeleton organization	3.24	0.012
**GOTERM_BP_FAT**	GO:0003002~regionalization	1.79	0.013
**GOTERM_BP_FAT**	GO:0045941~positive regulation of transcription	1.50	0.013
**KEGG_PATHWAY**	mmu04520:Adherens junction	2.46	0.013
**SP_PIR_KEYWORDS**	ubl conjugation pathway	1.49	0.013
**GOTERM_BP_FAT**	GO:0019941~modification-dependent protein catabolic process	1.48	0.014
**GOTERM_BP_FAT**	GO:0043632~modification-dependent macromolecule catabolic process	1.48	0.014
**GOTERM_CC_FAT**	GO:0000785~chromatin	1.91	0.017
**GOTERM_CC_FAT**	GO:0044429~mitochondrial part	1.47	0.017
**KEGG_PATHWAY**	mmu05210:Colorectal cancer	2.34	0.018
**SP_PIR_KEYWORDS**	protein transport	1.49	0.018
**GOTERM_BP_FAT**	GO:0009057~macromolecule catabolic process	1.41	0.021
**GOTERM_BP_FAT**	GO:0006606~protein import into nucleus	2.67	0.023
**SP_PIR_KEYWORDS**	atp-binding	1.27	0.024
**KEGG_PATHWAY**	mmu04722:Neurotrophin signaling pathway	2.03	0.026
**GOTERM_BP_FAT**	GO:0032886~regulation of microtubule-based process	2.88	0.027
**GOTERM_BP_FAT**	GO:0010628~positive regulation of gene expression	1.48	0.027
**SP_PIR_KEYWORDS**	mitochondrion	1.35	0.030
**GOTERM_CC_FAT**	GO:0005743~mitochondrial inner membrane	1.63	0.031
**KEGG_PATHWAY**	mmu04360:Axon guidance	2.01	0.031
**GOTERM_BP_FAT**	GO:0034621~cellular macromolecular complex subunit organization	1.70	0.033
**SP_PIR_KEYWORDS**	cell cycle	1.48	0.036
**GOTERM_BP_FAT**	GO:0010604~positive regulation of macromolecule metabolic process	1.40	0.037
**SP_PIR_KEYWORDS**	Proto-oncogene	2.22	0.040
**KEGG_PATHWAY**	mmu05220:Chronic myeloid leukemia	2.37	0.041
**GOTERM_BP_FAT**	GO:0051170~nuclear import	2.57	0.045
**GOTERM_CC_FAT**	GO:0000139~Golgi membrane	1.96	0.046
**GOTERM_BP_FAT**	GO:0001655~urogenital system development	1.91	0.050
**KEGG_PATHWAY**	mmu04916:Melanogenesis	2.15	0.052
**GOTERM_BP_FAT**	GO:0043933~macromolecular complex subunit organization	1.54	0.053
**GOTERM_BP_FAT**	GO:0008285~negative regulation of cell proliferation	1.71	0.056
**GOTERM_BP_FAT**	GO:0030163~protein catabolic process	1.42	0.058
**KEGG_PATHWAY**	mmu04350:TGF-beta signaling pathway	2.23	0.065
**GOTERM_BP_FAT**	GO:0065003~macromolecular complex assembly	1.56	0.069

Category	Term	Fold Enrichment[1]	FDR[2]
**UP_SEQ_FEATURE**	nucleotide phosphate-binding region:GTP	1.58	0.075
**GOTERM_CC_FAT**	GO:0005740~mitochondrial envelope	1.51	0.079
**GOTERM_BP_FAT**	GO:0001701~in utero embryonic development	1.63	0.080
**GOTERM_MF_FAT**	GO:0016563~transcription activator activity	1.64	0.082
**GOTERM_BP_FAT**	GO:0034622~cellular macromolecular complex assembly	1.70	0.084
**GOTERM_MF_FAT**	GO:0046914~transition metal ion binding	1.17	0.085
**GOTERM_BP_FAT**	GO:0007026~negative regulation of microtubule depolymerization	4.64	0.085
**GOTERM_BP_FAT**	GO:0031114~regulation of microtubule depolymerization	4.64	0.085
**SP_PIR_KEYWORDS**	zinc	1.20	0.087
**GOTERM_BP_FAT**	GO:0022402~cell cycle process	1.50	0.087
**GOTERM_MF_FAT**	GO:0046872~metal ion binding	1.13	0.090
**GOTERM_BP_FAT**	GO:0051603~proteolysis involved in cellular protein catabolic process	1.42	0.091
**GOTERM_BP_FAT**	GO:0046907~intracellular transport	1.48	0.092
**GOTERM_MF_FAT**	GO:0019899~enzyme binding	1.68	0.093
**GOTERM_MF_FAT**	GO:0004672~protein kinase activity	1.40	0.094
**GOTERM_CC_FAT**	GO:0019866~organelle inner membrane	1.57	0.10
**GOTERM_BP_FAT**	GO:0043069~negative regulation of programmed cell death	1.65	0.11
**GOTERM_BP_FAT**	GO:0043066~negative regulation of apoptosis	1.66	0.11
**KEGG_PATHWAY**	mmu04810:Regulation of actin cytoskeleton	1.69	0.12
**GOTERM_BP_FAT**	GO:0044257~cellular protein catabolic process	1.41	0.12
**GOTERM_MF_FAT**	GO:0003712~transcription cofactor activity	1.77	0.12
**GOTERM_MF_FAT**	GO:0043169~cation binding	1.12	0.12
**GOTERM_BP_FAT**	GO:0060548~negative regulation of cell death	1.64	0.12
**GOTERM_CC_FAT**	GO:0031966~mitochondrial membrane	1.51	0.12
**INTERPRO**	IPR001680:WD40 repeat	1.63	0.12
**GOTERM_BP_FAT**	GO:0022403~cell cycle phase	1.54	0.13
**GOTERM_BP_FAT**	GO:0006605~protein targeting	1.90	0.14
**GOTERM_BP_FAT**	GO:0048568~embryonic organ development	1.64	0.14
**GOTERM_MF_FAT**	GO:0000287~magnesium ion binding	1.48	0.14
**GOTERM_MF_FAT**	GO:0004674~protein serine/threonine kinase activity	1.47	0.15
**GOTERM_BP_FAT**	GO:0051960~regulation of nervous system development	1.84	0.16
**SP_PIR_KEYWORDS**	metal-binding	1.16	0.16
**GOTERM_CC_FAT**	GO:0019898~extrinsic to membrane	1.44	0.17
**GOTERM_CC_FAT**	GO:0042995~cell projection	1.39	0.18
**GOTERM_MF_FAT**	GO:0003723~RNA binding	1.36	0.18
**GOTERM_CC_FAT**	GO:0044451~nucleoplasm part	1.41	0.19
**GOTERM_BP_FAT**	GO:0043244~regulation of protein complex	2.72	0.20

Category	Term	Fold Enrichment[1]	FDR[2]
	disassembly		
**GOTERM_BP_FAT**	GO:0048598~embryonic morphogenesis	1.50	0.20
**UP_SEQ_FEATURE**	mutagenesis site	1.31	0.21
**GOTERM_MF_FAT**	GO:0043167~ion binding	1.12	0.21
**GOTERM_BP_FAT**	GO:0051254~positive regulation of RNA metabolic process	1.46	0.23
**GOTERM_BP_FAT**	GO:0051493~regulation of cytoskeleton organization	2.03	0.24
**GOTERM_BP_FAT**	GO:0010941~regulation of cell death	1.38	0.25
**GOTERM_BP_FAT**	GO:0031328~positive regulation of cellular biosynthetic process	1.39	0.25
**SP_PIR_KEYWORDS**	rna-binding	1.41	0.26
**GOTERM_BP_FAT**	GO:0042981~regulation of apoptosis	1.38	0.26
**GOTERM_CC_FAT**	GO:0070161~anchoring junction	1.91	0.27
**SP_PIR_KEYWORDS**	transferase	1.22	0.27
**GOTERM_BP_FAT**	GO:0035239~tube morphogenesis	1.75	0.28
**GOTERM_BP_FAT**	GO:0045893~positive regulation of transcription, DNA-dependent	1.45	0.28
**GOTERM_BP_FAT**	GO:0051494~negative regulation of cytoskeleton organization	2.57	0.29
**GOTERM_BP_FAT**	GO:0034613~cellular protein localization	1.54	0.29
**GOTERM_BP_FAT**	GO:0017038~protein import	2.14	0.30
**GOTERM_CC_FAT**	GO:0005815~microtubule organizing center	1.70	0.30
**GOTERM_BP_FAT**	GO:0043067~regulation of programmed cell death	1.38	0.30
**BIOCARTA**	m_tgfbPathway:TGF beta signaling pathway	3.37	0.33
**KEGG_PATHWAY**	mmu05215:Prostate cancer	2.08	0.33
**INTERPRO**	IPR015943:WD40/YVTN repeat-like	1.53	0.34
**GOTERM_BP_FAT**	GO:0070727~cellular macromolecule localization	1.53	0.36
**GOTERM_BP_FAT**	GO:0031111~negative regulation of microtubule polymerization or depolymerization	4.06	0.36
**GOTERM_BP_FAT**	GO:0009891~positive regulation of biosynthetic process	1.37	0.37
**INTERPRO**	IPR019775:WD40 repeat, conserved site	1.58	0.37
**GOTERM_BP_FAT**	GO:0000278~mitotic cell cycle	1.60	0.38
**GOTERM_CC_FAT**	GO:0005813~centrosome	1.76	0.38
**GOTERM_MF_FAT**	GO:0043565~sequence-specific DNA binding	1.38	0.38
**GOTERM_BP_FAT**	GO:0010557~positive regulation of macromolecule biosynthetic process	1.38	0.39
**GOTERM_MF_FAT**	GO:0003713~transcription coactivator activity	2.00	0.41
**UP_SEQ_FEATURE**	compositionally biased region:Poly-Ser	1.43	0.42
**SP_PIR_KEYWORDS**	wd repeat	1.55	0.42
**GOTERM_BP_FAT**	GO:0006796~phosphate metabolic process	1.29	0.43
**GOTERM_BP_FAT**	GO:0006793~phosphorus metabolic process	1.29	0.43

Category	Term	Fold Enrichment[1]	FDR[2]
**GOTERM_BP_FAT**	GO:0006928~cell motion	1.47	0.44
**SP_PIR_KEYWORDS**	kinase	1.32	0.44
**SP_PIR_KEYWORDS**	serine/threonine-protein kinase	1.44	0.45
**GOTERM_BP_FAT**	GO:0010639~negative regulation of organelle organization	2.30	0.45
**GOTERM_BP_FAT**	GO:0016310~phosphorylation	1.32	0.45
**GOTERM_CC_FAT**	GO:0005874~microtubule	1.59	0.47
**GOTERM_BP_FAT**	GO:0001656~metanephros development	2.35	0.48
**SP_PIR_KEYWORDS**	coiled coil	1.19	0.49
**GOTERM_BP_FAT**	GO:0035282~segmentation	2.40	0.50
**INTERPRO**	IPR019781:WD40 repeat, subgroup	1.60	0.53
**GOTERM_CC_FAT**	GO:0005783~endoplasmic reticulum	1.29	0.54
**GOTERM_BP_FAT**	GO:0010648~negative regulation of cell communication	1.67	0.56
**GOTERM_BP_FAT**	GO:0044092~negative regulation of molecular function	1.82	0.58
**INTERPRO**	IPR005225:Small GTP-binding protein	1.73	0.60
**SP_PIR_KEYWORDS**	methylation	1.59	0.67
**SP_PIR_KEYWORDS**	wnt signaling pathway	1.84	0.70
**GOTERM_BP_FAT**	GO:0046822~regulation of nucleocytoplasmic transport	2.78	0.71
**GOTERM_BP_FAT**	GO:0043242~negative regulation of protein complex disassembly	2.78	0.71
**KEGG_PATHWAY**	mmu05211:Renal cell carcinoma	2.18	0.72
**GOTERM_BP_FAT**	GO:0008045~motor axon guidance	4.17	0.73
**GOTERM_BP_FAT**	GO:0006468~protein amino acid phosphorylation	1.33	0.73
**GOTERM_BP_FAT**	GO:0009953~dorsal/ventral pattern formation	2.23	0.73
**GOTERM_CC_FAT**	GO:0005856~cytoskeleton	1.24	0.75
**GOTERM_CC_FAT**	GO:0043005~neuron projection	1.56	0.81
**SP_PIR_KEYWORDS**	ligase	1.49	0.82
**GOTERM_BP_FAT**	GO:0033043~regulation of organelle organization	1.73	0.84
**GOTERM_BP_FAT**	GO:0000279~M phase	1.51	0.86
**SP_PIR_KEYWORDS**	mitochondrion inner membrane	1.64	0.89
**GOTERM_BP_FAT**	GO:0001763~morphogenesis of a branching structure	1.82	0.89
**KEGG_PATHWAY**	mmu05218:Melanoma	2.15	0.89
**GOTERM_MF_FAT**	GO:0016564~transcription repressor activity	1.61	0.89
**PIR_SUPERFAMILY**	PIRSF001711:ADP-ribosylation factor	3.18	0.92
**GOTERM_CC_FAT**	GO:0005643~nuclear pore	2.27	0.93
**GOTERM_CC_FAT**	GO:0005912~adherens junction	1.90	0.93
**KEGG_PATHWAY**	mmu04144:Endocytosis	1.61	0.97
**GOTERM_BP_FAT**	GO:0044093~positive regulation of molecular function	1.48	1.01
**INTERPRO**	IPR008271:Serine/threonine protein kinase,	1.45	1.02

Category	Term	Fold Enrichment[1]	FDR[2]
	active site		
**GOTERM_BP_FAT**	GO:0045664~regulation of neuron differentiation	1.91	1.03
**GOTERM_BP_FAT**	GO:0033554~cellular response to stress	1.41	1.03
**GOTERM_BP_FAT**	GO:0006396~RNA processing	1.40	1.04
**GOTERM_BP_FAT**	GO:0051129~negative regulation of cellular component organization	1.95	1.10
**GOTERM_MF_FAT**	GO:0019904~protein domain specific binding	1.63	1.14
**GOTERM_MF_FAT**	GO:0016881~acid-amino acid ligase activity	1.68	1.14
**GOTERM_BP_FAT**	GO:0051252~regulation of RNA metabolic process	1.19	1.14
**GOTERM_MF_FAT**	GO:0019992~diacylglycerol binding	2.25	1.14
**GOTERM_BP_FAT**	GO:0007411~axon guidance	1.92	1.17
**SMART**	SM00320:WD40	1.48	1.17
**GOTERM_BP_FAT**	GO:0007507~heart development	1.57	1.19
**GOTERM_MF_FAT**	GO:0060589~nucleoside-triphosphatase regulator activity	1.43	1.22
**UP_SEQ_FEATURE**	short sequence motif:Nuclear localization signal	1.45	1.29
**KEGG_PATHWAY**	mmu05223:Non-small cell lung cancer	2.31	1.30
**INTERPRO**	IPR017970:Homeobox, conserved site	1.57	1.31
**SP_PIR_KEYWORDS**	transit peptide	1.37	1.33
**GOTERM_BP_FAT**	GO:0048678~response to axon injury	5.05	1.35
**GOTERM_CC_FAT**	GO:0030054~cell junction	1.37	1.35
**SP_PIR_KEYWORDS**	magnesium	1.39	1.39
**INTERPRO**	IPR011989:Armadillo-like helical	1.77	1.40
**GOTERM_BP_FAT**	GO:0009952~anterior/posterior pattern formation	1.70	1.45
**GOTERM_BP_FAT**	GO:0032880~regulation of protein localization	1.95	1.49
**UP_SEQ_FEATURE**	splice variant	1.09	1.53
**INTERPRO**	IPR012677:Nucleotide-binding, alpha-beta plait	1.58	1.55
**GOTERM_BP_FAT**	GO:0042127~regulation of cell proliferation	1.34	1.58
**INTERPRO**	IPR017441:Protein kinase, ATP binding site	1.37	1.68
**KEGG_PATHWAY**	mmu00510:N-Glycan biosynthesis	2.41	1.69
**GOTERM_BP_FAT**	GO:0007265~Ras protein signal transduction	2.20	1.76
**GOTERM_BP_FAT**	GO:0070085~glycosylation	1.93	1.79
**GOTERM_BP_FAT**	GO:0043413~biopolymer glycosylation	1.93	1.79
**GOTERM_BP_FAT**	GO:0006486~protein amino acid glycosylation	1.93	1.79
**GOTERM_BP_FAT**	GO:0021953~central nervous system neuron differentiation	2.47	1.80
**GOTERM_MF_FAT**	GO:0004842~ubiquitin-protein ligase activity	1.85	1.90
**SP_PIR_KEYWORDS**	nucleotide binding	2.09	2.00
**GOTERM_BP_FAT**	GO:0006355~regulation of transcription, DNA-dependent	1.19	2.00

Category	Term	Fold Enrichment[1]	FDR[2]
**GOTERM_BP_FAT**	GO:0006886~intracellular protein transport	1.48	2.06
**SP_PIR_KEYWORDS**	microtubule	1.53	2.08
**GOTERM_BP_FAT**	GO:0009968~negative regulation of signal transduction	1.63	2.08
**GOTERM_CC_FAT**	GO:0030173~integral to Golgi membrane	2.73	2.09
**GOTERM_CC_FAT**	GO:0031228~intrinsic to Golgi membrane	2.73	2.09
**GOTERM_BP_FAT**	GO:0045944~positive regulation of transcription from RNA polymerase II promoter	1.41	2.14
**SP_PIR_KEYWORDS**	Chaperone	1.66	2.15
**GOTERM_MF_FAT**	GO:0019787~small conjugating protein ligase activity	1.76	2.22
**GOTERM_BP_FAT**	GO:0045596~negative regulation of cell differentiation	1.60	2.27
**GOTERM_BP_FAT**	GO:0043085~positive regulation of catalytic activity	1.49	2.29
**GOTERM_MF_FAT**	GO:0016879~ligase activity, forming carbon-nitrogen bonds	1.59	2.33
**KEGG_PATHWAY**	mmu05213:Endometrial cancer	2.27	2.35
**GOTERM_BP_FAT**	GO:0016477~cell migration	1.51	2.36
**INTERPRO**	IPR017986:WD40 repeat, region	1.53	2.46
**SP_PIR_KEYWORDS**	P-loop	2.06	2.46
**GOTERM_BP_FAT**	GO:0009100~glycoprotein metabolic process	1.68	2.49
**GOTERM_BP_FAT**	GO:0022613~ribonucleoprotein complex biogenesis	1.71	2.51
**GOTERM_CC_FAT**	GO:0046930~pore complex	2.02	2.52
**GOTERM_BP_FAT**	GO:0051340~regulation of ligase activity	5.56	2.53
**GOTERM_BP_FAT**	GO:0006338~chromatin remodeling	2.60	2.55
**GOTERM_BP_FAT**	GO:0007167~enzyme linked receptor protein signaling pathway	1.47	2.57
**GOTERM_BP_FAT**	GO:0032259~methylation	1.97	2.59
**INTERPRO**	IPR000719:Protein kinase, core	1.34	2.67
**UP_SEQ_FEATURE**	cross-link:Glycyl lysine isopeptide (Lys-Gly) (interchain with G-Cter in ubiquitin)	1.58	2.68
**SP_PIR_KEYWORDS**	kinetochore	2.09	2.71
**GOTERM_BP_FAT**	GO:0043623~cellular protein complex assembly	1.80	2.75
**GOTERM_BP_FAT**	GO:0051174~regulation of phosphorus metabolic process	1.44	2.80
**GOTERM_BP_FAT**	GO:0019220~regulation of phosphate metabolic process	1.44	2.80
**INTERPRO**	IPR001806:Ras GTPase	1.72	2.85
**GOTERM_CC_FAT**	GO:0000790~nuclear chromatin	2.20	2.89
**GOTERM_MF_FAT**	GO:0030695~GTPase regulator activity	1.40	2.92
**GOTERM_BP_FAT**	GO:0021522~spinal cord motor neuron differentiation	3.24	2.93
**GOTERM_BP_FAT**	GO:0016458~gene silencing	2.16	3.03
**GOTERM_BP_FAT**	GO:0070271~protein complex biogenesis	1.52	3.17

Category	Term	Fold Enrichment[1]	FDR[2]
**GOTERM_BP_FAT**	GO:0006461~protein complex assembly	1.52	3.17
**SP_PIR_KEYWORDS**	cell junction	1.36	3.18
**GOTERM_BP_FAT**	GO:0006333~chromatin assembly or disassembly	1.79	3.20
**GOTERM_CC_FAT**	GO:0005819~spindle	1.78	3.21
**GOTERM_BP_FAT**	GO:0043414~biopolymer methylation	2.01	3.28
**GOTERM_CC_FAT**	GO:0005730~nucleolus	1.43	3.28
**GOTERM_BP_FAT**	GO:0060541~respiratory system development	1.73	3.29
**GOTERM_MF_FAT**	GO:0003924~GTPase activity	1.71	3.34
**GOTERM_BP_FAT**	GO:0051336~regulation of hydrolase activity	1.56	3.40
**GOTERM_BP_FAT**	GO:0048609~reproductive process in a multicellular organism	1.36	3.56
**GOTERM_BP_FAT**	GO:0032504~multicellular organism reproduction	1.36	3.56
**KEGG_PATHWAY**	mmu04340:Hedgehog signaling pathway	2.18	3.60
**GOTERM_BP_FAT**	GO:0032583~regulation of gene-specific transcription	2.03	3.69
**GOTERM_BP_FAT**	GO:0001944~vasculature development	1.48	3.70
**GOTERM_BP_FAT**	GO:0006259~DNA metabolic process	1.36	3.77
**GOTERM_BP_FAT**	GO:0048732~gland development	1.55	3.78
**KEGG_PATHWAY**	mmu05214:Glioma	2.06	3.82
**GOTERM_MF_FAT**	GO:0017124~SH3 domain binding	1.94	3.84
**GOTERM_BP_FAT**	GO:0048562~embryonic organ morphogenesis	1.61	3.98
**GOTERM_CC_FAT**	GO:0000228~nuclear chromosome	1.72	4.09
**GOTERM_CC_FAT**	GO:0044456~synapse part	1.52	4.10
**SP_PIR_KEYWORDS**	molecular chaperone	3.64	4.11
**GOTERM_BP_FAT**	GO:0016569~covalent chromatin modification	1.83	4.11
**GOTERM_BP_FAT**	GO:0051094~positive regulation of developmental process	1.52	4.24
**GOTERM_BP_FAT**	GO:0001756~somitogenesis	2.37	4.26
**GOTERM_CC_FAT**	GO:0045202~synapse	1.41	4.26
**GOTERM_BP_FAT**	GO:0007423~sensory organ development	1.46	4.28
**GOTERM_BP_FAT**	GO:0030900~forebrain development	1.59	4.38
**KEGG_PATHWAY**	mmu05217:Basal cell carcinoma	2.14	4.40
**GOTERM_BP_FAT**	GO:0042325~regulation of phosphorylation	1.43	4.42
**KEGG_PATHWAY**	mmu03018:RNA degradation	2.08	4.52
**SP_PIR_KEYWORDS**	tpr repeat	1.63	4.58
**GOTERM_BP_FAT**	GO:0001837~epithelial to mesenchymal transition	3.71	4.67
**GOTERM_BP_FAT**	GO:0060688~regulation of morphogenesis of a branching structure	2.86	4.72
**GOTERM_BP_FAT**	GO:0009954~proximal/distal pattern formation	2.86	4.72
**INTERPRO**	IPR017442:Serine/threonine protein kinase-related	1.37	4.76

Category	Term	Fold Enrichment[1]	FDR[2]
**GOTERM_BP_FAT**	GO:0000087~M phase of mitotic cell cycle	1.54	4.79
**INTERPRO**	IPR011016:Zinc finger, RING-CH-type	4.19	4.91
**INTERPRO**	IPR017967:HMG box A DNA-binding domain, conserved site	4.19	4.91
**GOTERM_BP_FAT**	GO:0003006~reproductive developmental process	1.45	4.93
**GOTERM_BP_FAT**	GO:0006730~one-carbon metabolic process	1.72	4.95
**GOTERM_BP_FAT**	GO:0009101~glycoprotein biosynthetic process	1.72	4.95

Note:
1. Fold enrichment characterizes the ratio between the percentage of motif target genes that belong to the gene set in question and the percentage of all genes in the genome that belong to the gene set in question.
2. In the FDR column, 2 means FDR = 2%, and 0.2 means FDR = 0.2%.

Supplementary Table S7. Number of annotated target genes of the new motif using different target gene definitions.

Motif mapping LR	TSS up (bp)	Cluster window (bp)	No. of sites in cluster window	Gene No. (All sites)	Gene No. (Clusters)
**Human**					
500	1000	500	≥ 2	11027	8651
1000	1000	500	≥ 2	9660	7205
2000	1000	500	≥ 2	8452	5966
1000	500	500	≥ 2	8718	6899
1000	1500	500	≥ 2	10113	7344
1000	2000	500	≥ 2	10457	7442
1000	1000	250	≥ 2	9660	6001
1000	1000	1000	≥ 2	9660	8046
1000	1000	250	≥ 3		3116
1000	1000	500	≥ 3		4832
1000	1000	1000	≥ 3		6182
**Mouse**					
500	1000	500	≥ 2	9289	7266
1000	1000	500	≥ 2	8121	6088
2000	1000	500	≥ 2	6998	4957
1000	500	500	≥ 2	7355	5868
1000	1500	500	≥ 2	8545	6160
1000	2000	500	≥ 2	8962	6253
1000	1000	250	≥ 2	8121	4960
1000	1000	1000	≥ 2	8121	6726
1000	1000	250	≥ 3		2502
1000	1000	500	≥ 3		4008
1000	1000	1000	≥ 3		5058

Motif	Enrichment level	Logo
Novel Motif	2.36	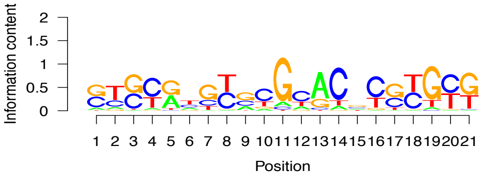
GLI	6.36	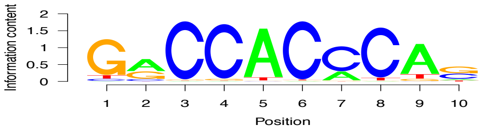
SP1	2.36	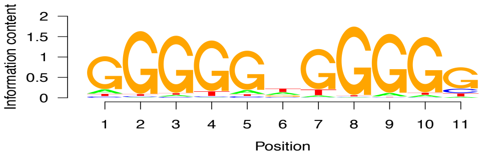
De novo class 1(1)	2.45	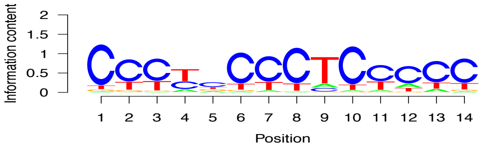
De novo class 1(2)	2.58	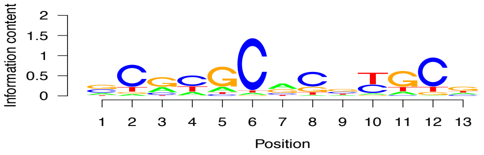
De novo class 1(3)	2.05	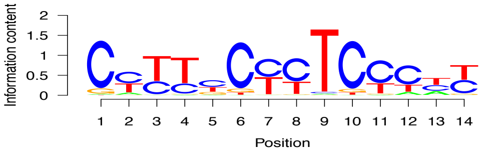
De novo class 2(1)	1.22	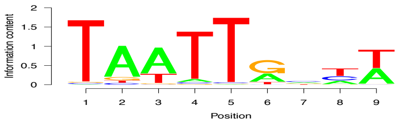
De novo class 2(2)	1.26	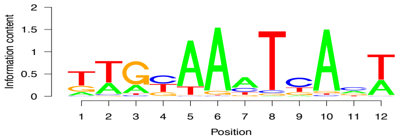
De novo class 2(3)	1.27	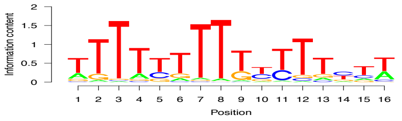

Supplementary Figure S1. Motifs discovered from GLI3 binding regions. For each motif, the motif logo and relative enrichment level (see Methods) are shown. The first six motifs passed the two-fold enrichment cutoff.

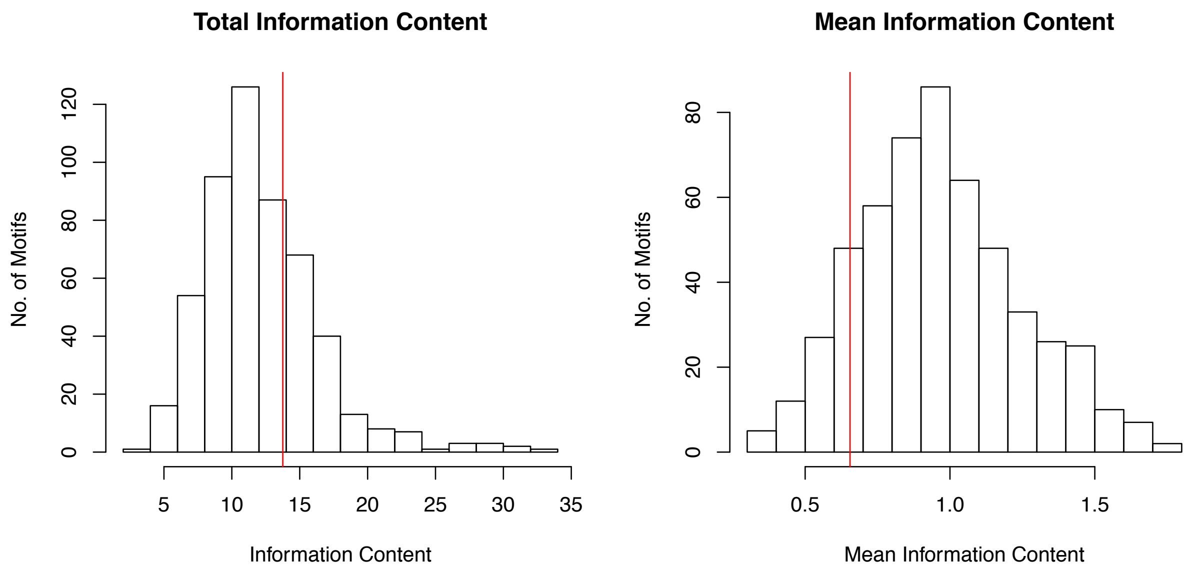

Supplementary Figure S2. Information content distribution for known motifs in TRANSFAC. The left plot shows the total information content. The right plot shows the per-nucleotide information content. Information content of the novel motif is indicated by the red vertical line in each plot.

Motif	Source	Logo
GLI	TRANSFAC: M01037	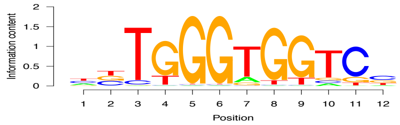
CTCF	JASPAR: MA0139.1	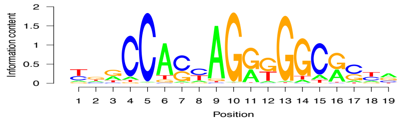
NRSF	TRANSFAC: M00256	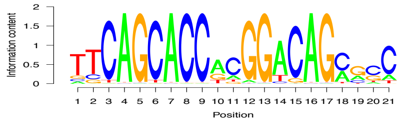
FOXA1	JASPAR: MA0148.1	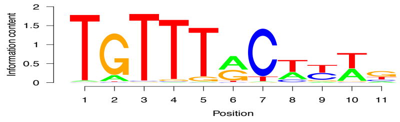
MYC	TRANSFAC: M00799	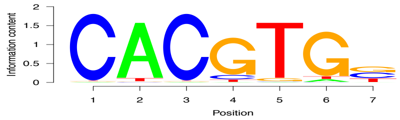
ER	TRANSFAC: M00959	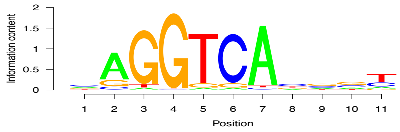
Oct4Sox2	Li et al. *Bioinformatics* (2007) 23: 1188-1194	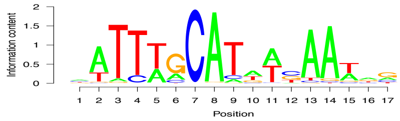
SP1	TRANSFAC: M00931	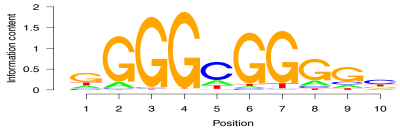

Supplementary Figure S3. The list and sequence logos of known motifs used in this study.

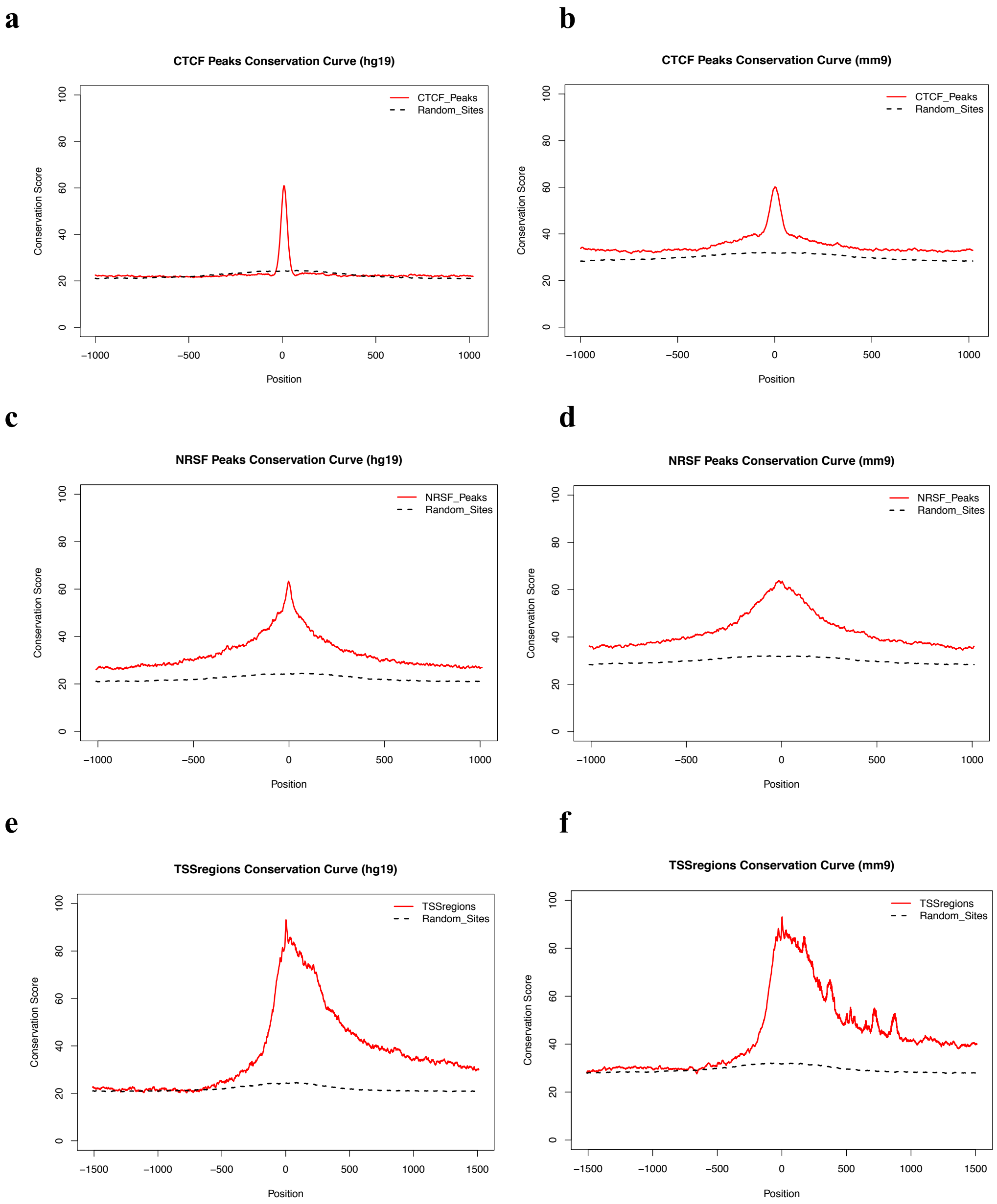

Supplementary Figure S4. Examples illustrating the ability of phastCons scores to describe sequence conservation. (a)-(d) phastCons conservation for transcription factor binding sites experimentally identified by ChIP-seq. Since many of these binding sites are functional, they are expected to be more conserved than random genomic sites on average. Therefore, they can be used as positive controls to test the ability of phastCons scores to characterize phylogenetic conservation given the presence of alignment uncertainties which may affect phastCons scores. Peak lists of four ChIP-seq experiments were obtained from ENCODE [2,3], including (a) CTCF in HepG2 cell line (human hg19, Uniform TFBS, 46,448 peaks), (b) CTCF in C2C12 cell line (mouse mm9, Caltech, 21,117 peaks), (c) NRSF in A549 cell line (human hg19, Uniform TFBS, 11,970 peaks), (d) NRSF in C2C12 cell line (mouse mm9, Caltech, 15,127). For each ChIP-seq peak list, the mean phastCons scores for the peak center and its flanking positions across all peaks are shown. Similarly, phastCons scores for random genomic sites are also shown. These figures show that phastCons scores for TF binding sites are higher than random genomic sites. Also, the scores of TF binding sites decay as one moves away from the binding center. These all indicate that phastCons scores used by us are able to characterize the average conservation of many genomic sites. (e)-(f)

phastCons conservation for transcription start sites (TSS) and its flanking regions in human (e) and mouse (f). In each species, mean phastCons score at each position was computed across all TSSs. The coordinate zero in the x-axis corresponds to TSS. Positive coordinates (right from 0) represent positions 3' downstream of TSS (i.e., first exon and sequences behind). Negative coordinates (left from 0) represent positions 5' upstream of TSS. These figures show that sequences around TSS are more conserved than random genomic sites. Also, regions 3' downstream of TSS (exons) are more conserved than regions 5' upstream of TSS, consistent with the strong selection pressure on exons. The analyses again illustrate that phastCons scores used by us are able to characterize the conservation despite the potential presence of alignment uncertainties.

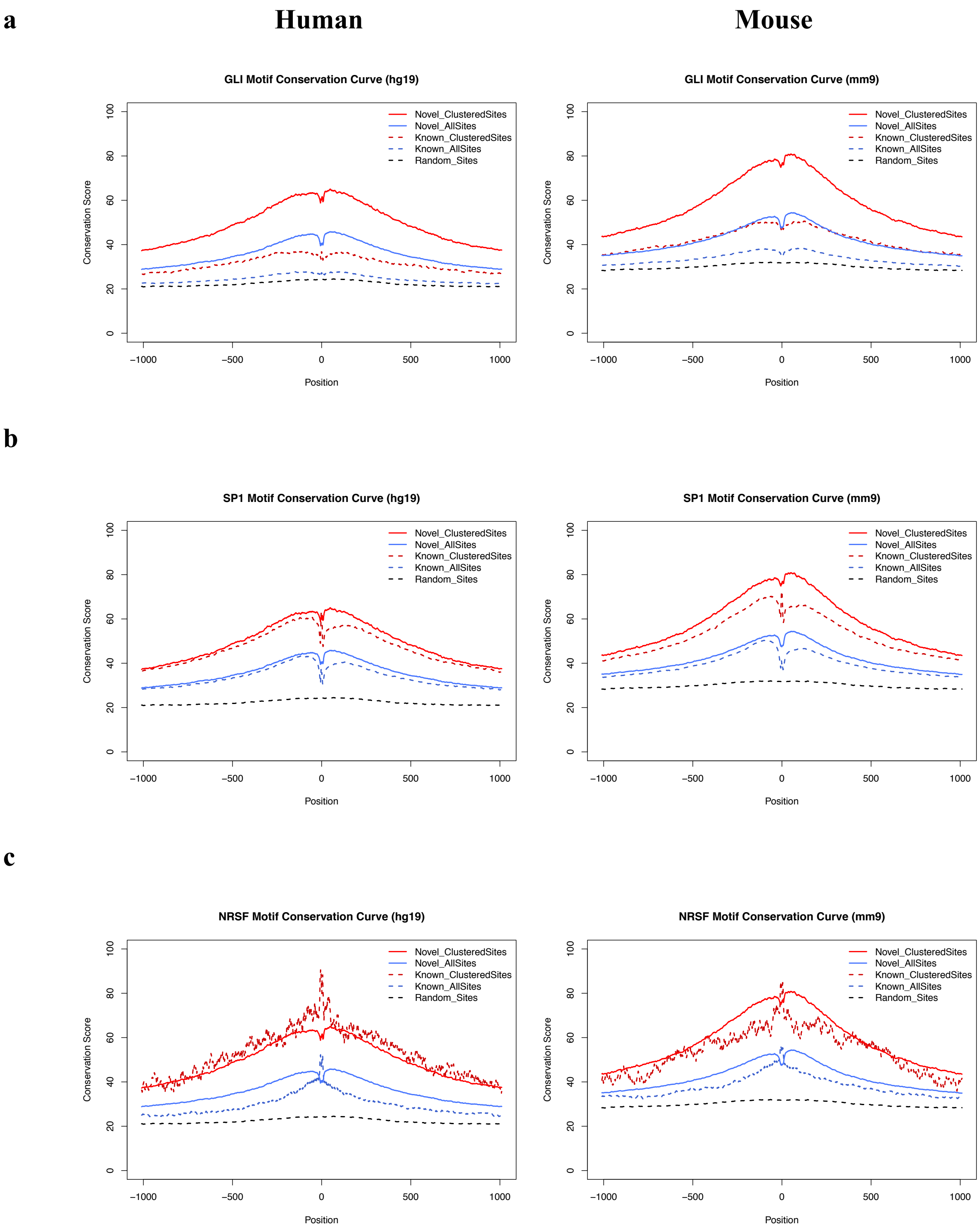

Supplementary Figure S5. Comparisons of the phylogenetic conservation between the new motif and several known motifs. The mean phastCons scores for each motif and its flanking positions in human (left) and mouse (right) genomes are shown. Position 0 corresponds to the motif center. Within each genome, the scores at each position are averaged across all loci in each of the five categories: clustered motif sites of the new motif, all motif sites of the new motif, clustered motif sites of the known motif, all motif sites of the known motif, and random genomic sites. (a) Comparison with the known GLI motif. (b) Comparison with the known SP1 motif. (c) Comparison with the known NRSF motif.

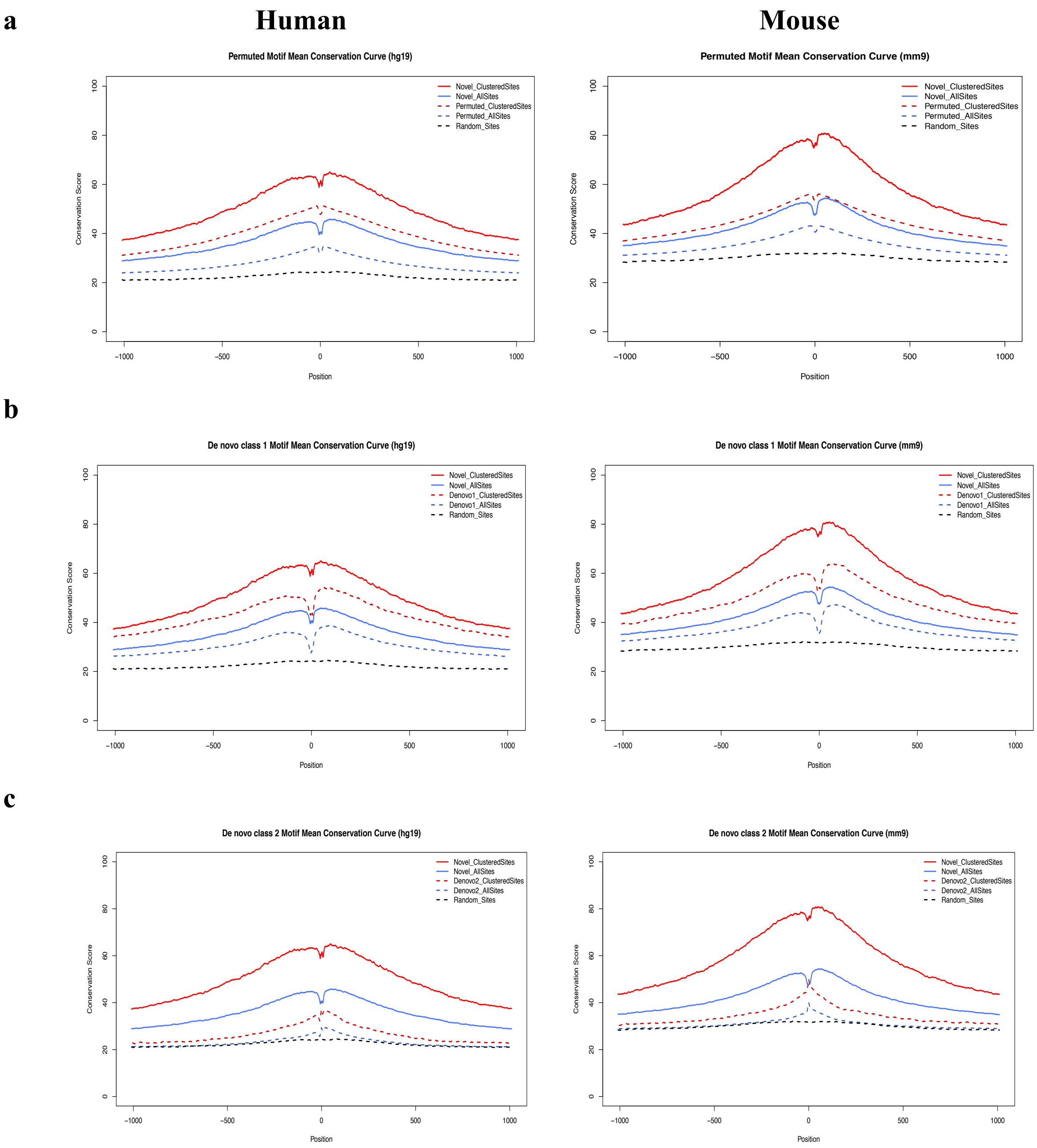

Supplementary Figure S6. Comparisons of the phylogenetic conservation between the new motif and the permuted, *de novo* class 1 and *de novo* class 2 motifs. The mean phastCons score curve for each motif and its flanking positions in human (left) and mouse (right) genomes is computed by averaging across all motif sites. The curves of all motifs in each motif category are then averaged. The average curves are shown for each motif category for clustered motif sites, all motif sites, and random genomic sites. Position 0 corresponds to the motif center. (a) Comparison with permuted motifs. (b) Comparison with *de novo* class 1 motifs. (c) Comparison with *de novo* class 2 motifs.

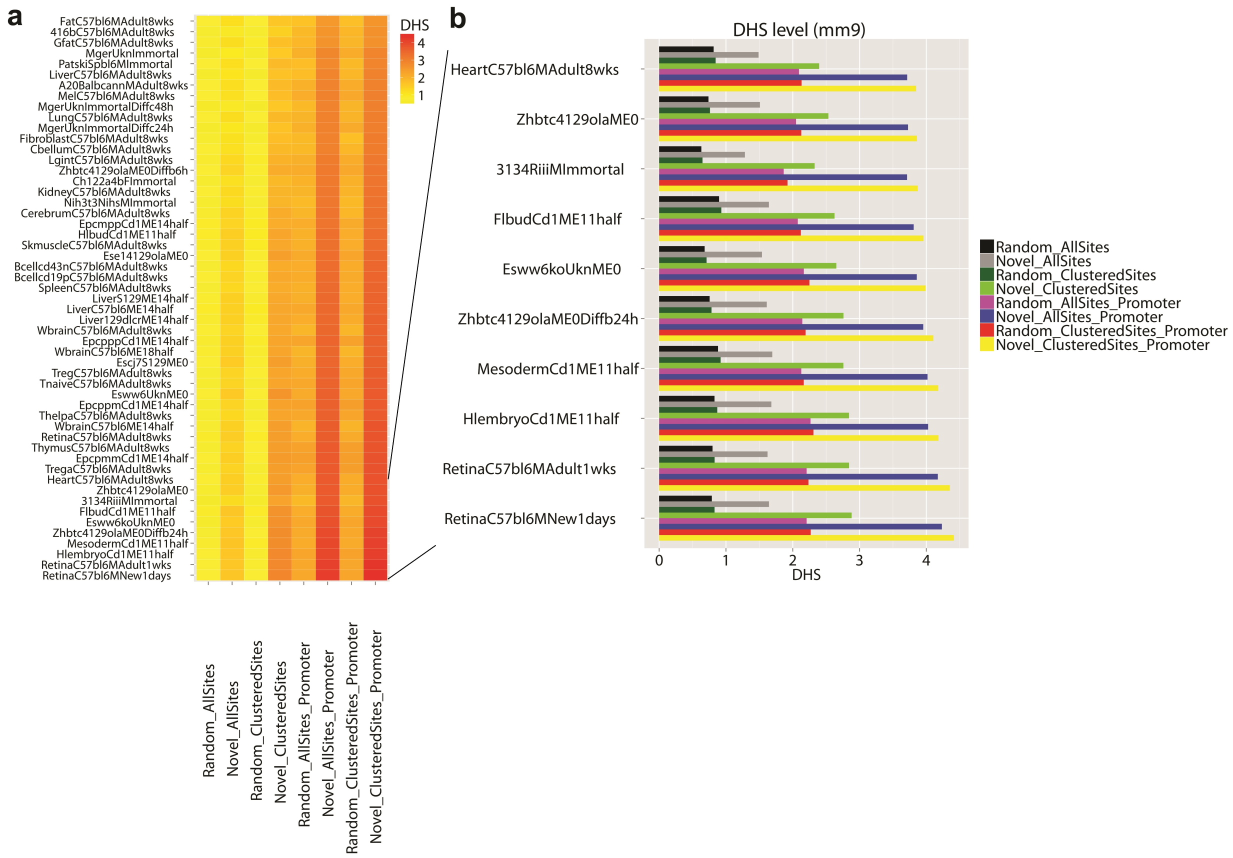

Supplementary Figure S7. DNase I hypersensitivity for different motif site categories in 53 mouse ENCODE cell lines. The motif site categories shown in the figure include all motif sites (Novel_AllSites), random control sites (Random_AllSites), clustered motif sites (Novel_ClusteredSites), random clustered sites (Random_ClusteredSites), all motif sites in promoters (Novel_AllSites_Promoter), random control sites in promoters (Random_AllSites_Promoter), clustered motif sites in promoters (Novel_ClusteredSites_Promoter), and random clustered sites in promoters (Random_ClusteredSites_Promoter). For each motif site category and cell line, the average DHS across all motif sites is shown. (a) Heatmap of DHS of different motif site categories in all cell lines. (b) Zoomed-in plot for 10 cell lines from the bottom of (a).

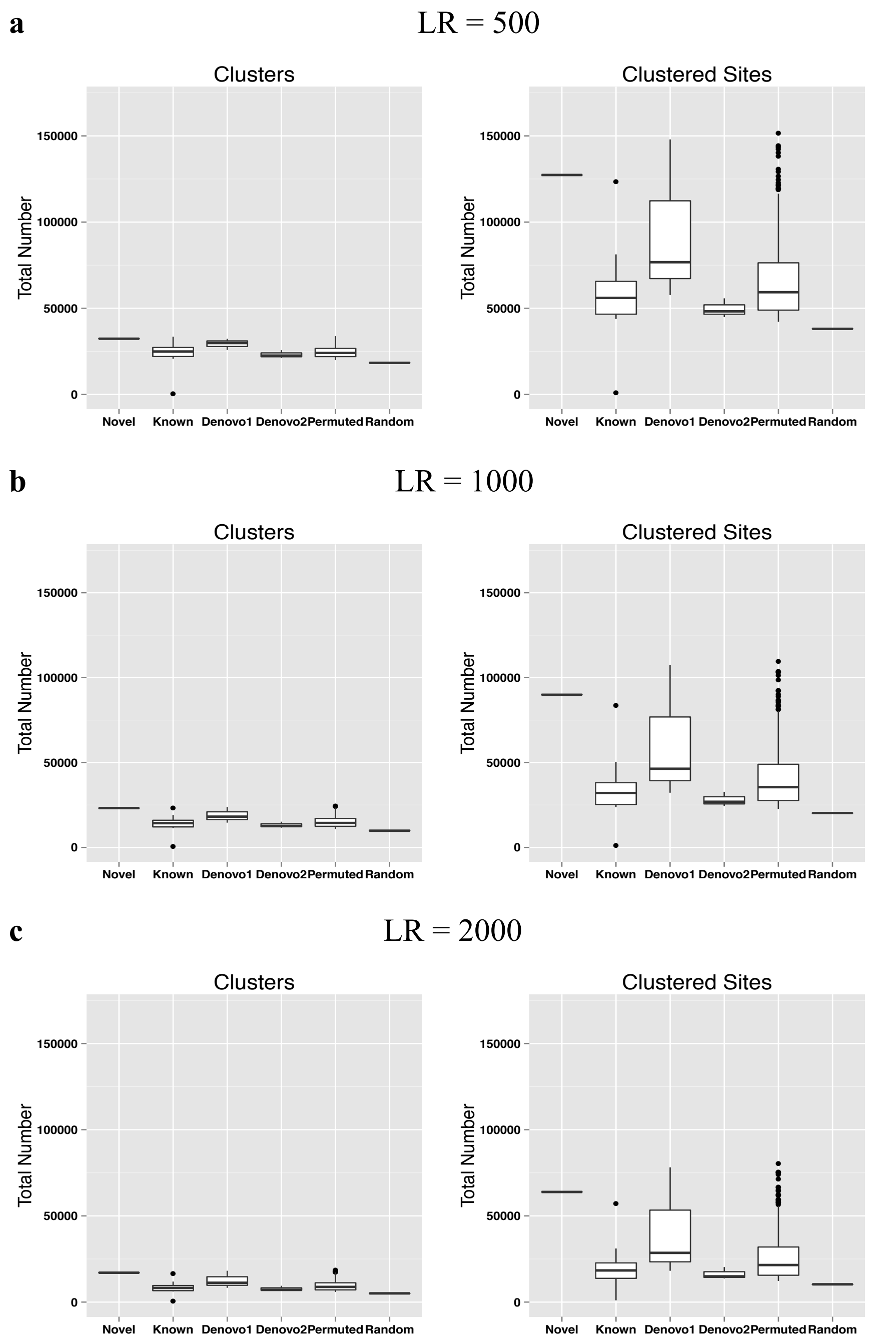

Supplementary Figure S8. Number of motif site clusters and clustered motif sites in human using different motif mapping criteria. (a) Motif mapping likelihood ratio ≥ 500 for the new motif. (b) Likelihood ratio ≥ 1000. (c) Likelihood ratio ≥ 2000. The number of motif site clusters (left) and clustered motif sites (right) are computed for each motif. Distributions of these numbers across all motifs are shown for the novel motif ("Novel"), eight known motifs ("Known"), three *de novo* class 1 motifs ("Denovo1"), three *de novo* class 2 motifs ("Denovo2"), 500 permuted motifs ("Permuted"), and random genomic control sites ("Random"). Motif site cluster is defined using 500bp window.

a LR = 500

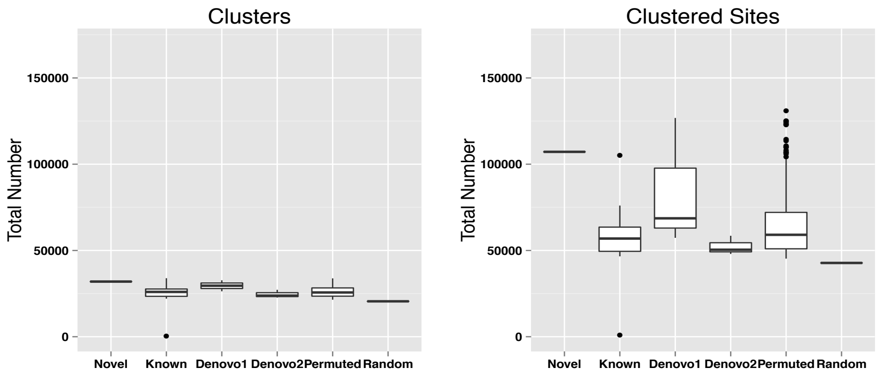

b LR = 1000

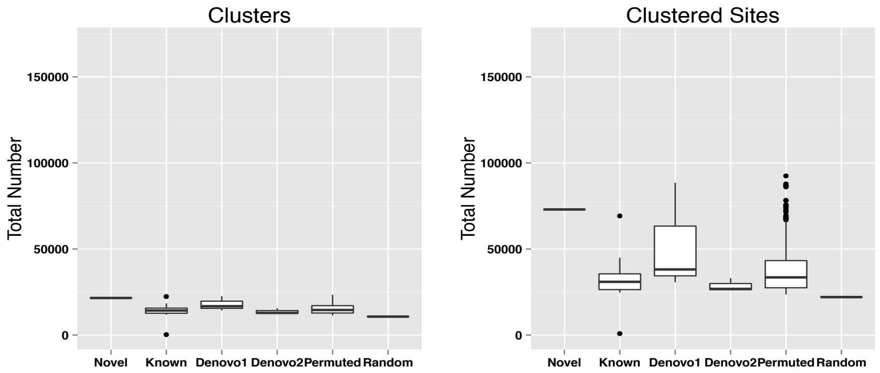

c LR = 2000

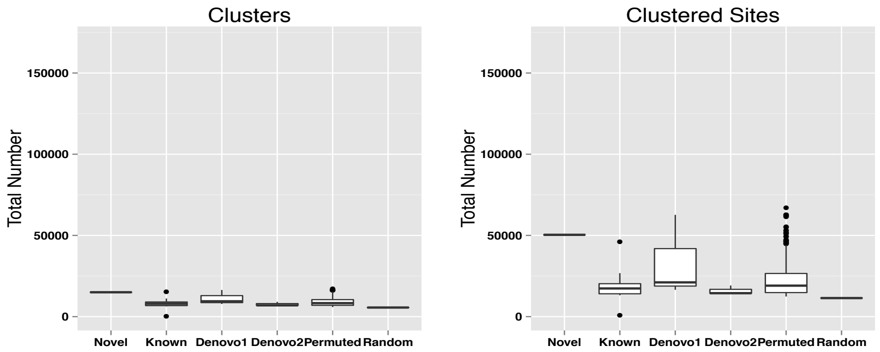

Supplementary Figure S9. Number of motif site clusters and clustered motif sites in mouse using different motif mapping criteria. (a) Motif mapping likelihood ratio ≥ 500 for the new motif. (b) Likelihood ratio ≥ 1000. (c) Likelihood ratio ≥ 2000. The number of motif site clusters (left) and clustered motif sites (right) are computed for each motif. Distributions of these numbers across all motifs are shown for the novel motif ("Novel"), eight known motifs ("Known"), three *de novo* class 1 motifs ("Denovo1"), three *de novo* class 2 motifs ("Denovo2"), 500 permuted motifs ("Permuted"), and random genomic control sites ("Random"). Motif site cluster is defined using 500bp window.

a Cluster window size = 250

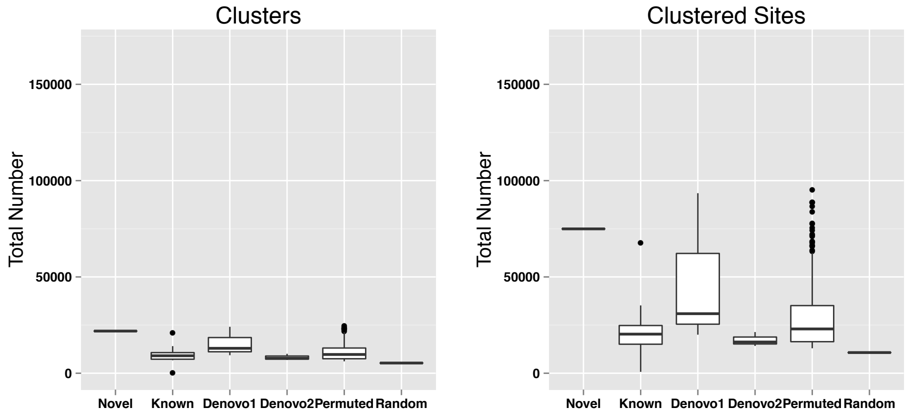

b Cluster window size = 1000

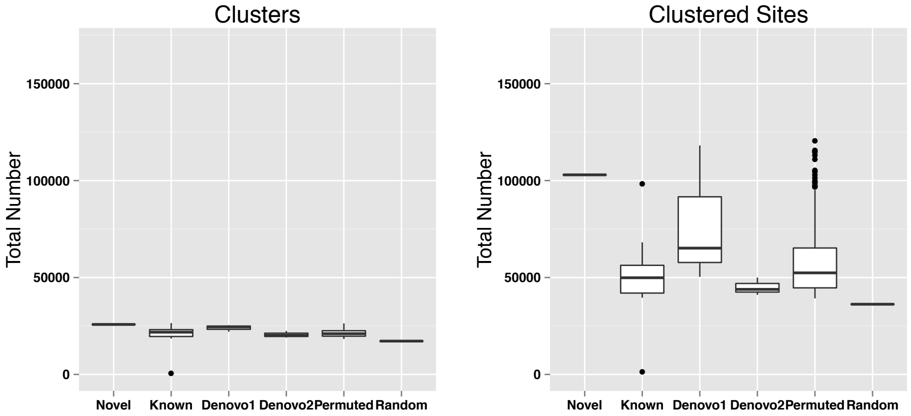

Supplementary Figure S10. Number of motif site clusters and clustered motif sites in human using different window size to define motif site clusters. (a) Window size = 250bp (i.e., any two motif sites separated by less than 250bp are merged). (b) Window size = 1000bp. The number of motif site clusters (left) and clustered motif sites (right) are computed for each motif. Distributions of these numbers across all motifs are shown for the novel motif ("Novel"), eight known motifs ("Known"), three *de novo* class 1 motifs ("Denovo1"), three *de novo* class 2 motifs ("Denovo2"), 500 permuted motifs ("Permuted"), and random genomic control sites ("Random"). Motif mapping likelihood ratio ≥ 1000 for the new motif.

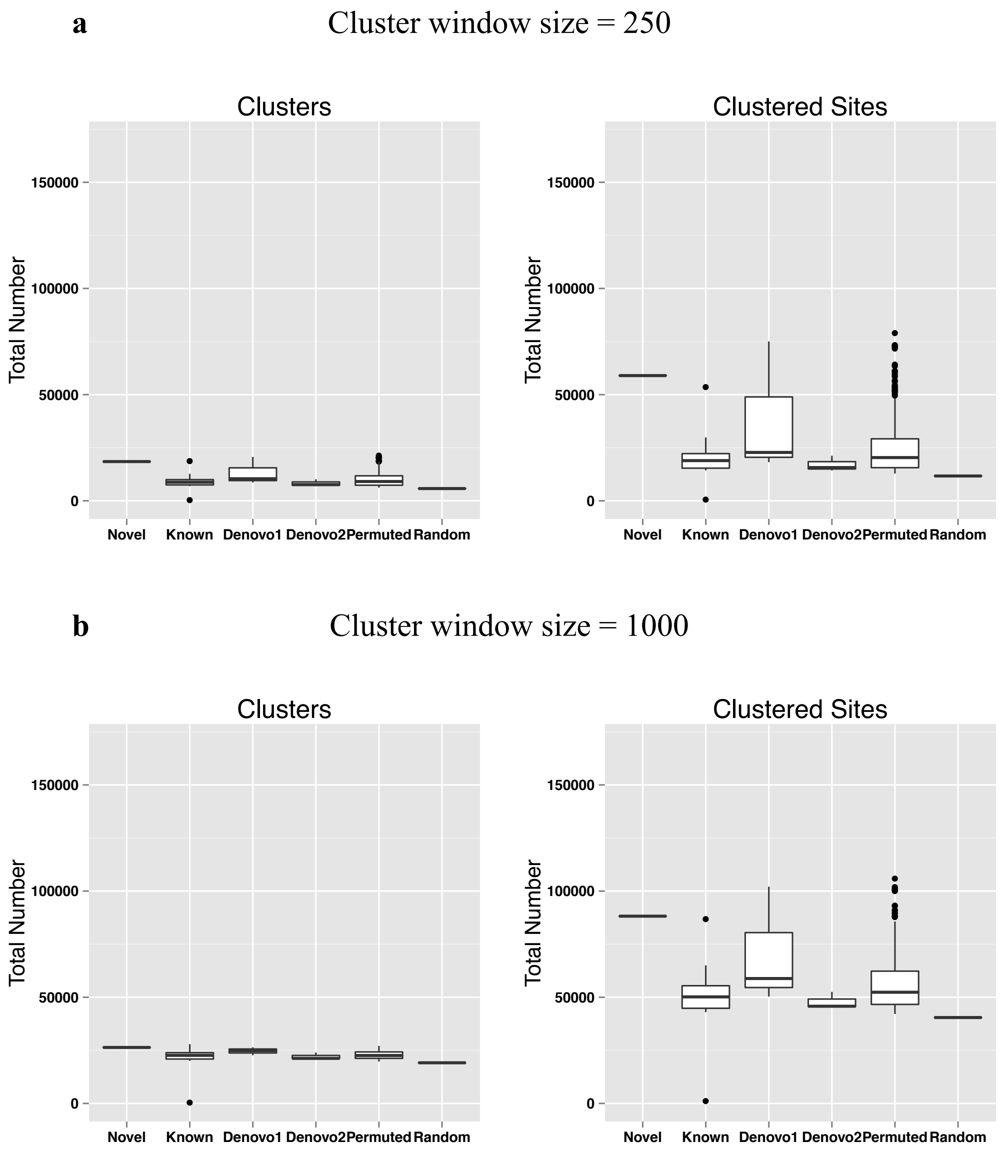

Supplementary Figure S11. Number of motif site clusters and clustered motif sites in mouse using different window size to define motif site clusters. (a) Window size = 250bp. (b) Window size = 1000bp. The number of motif site clusters (left) and clustered motif sites (right) are computed for each motif. Distributions of these numbers across all motifs are shown for the novel motif ("Novel"), eight known motifs ("Known"), three *de novo* class 1 motifs ("Denovo1"), three *de novo* class 2 motifs ("Denovo2"), 500 permuted motifs ("Permuted"), and random genomic control sites ("Random"). Motif mapping likelihood ratio ≥ 1000 for the new motif.

a LR = 500

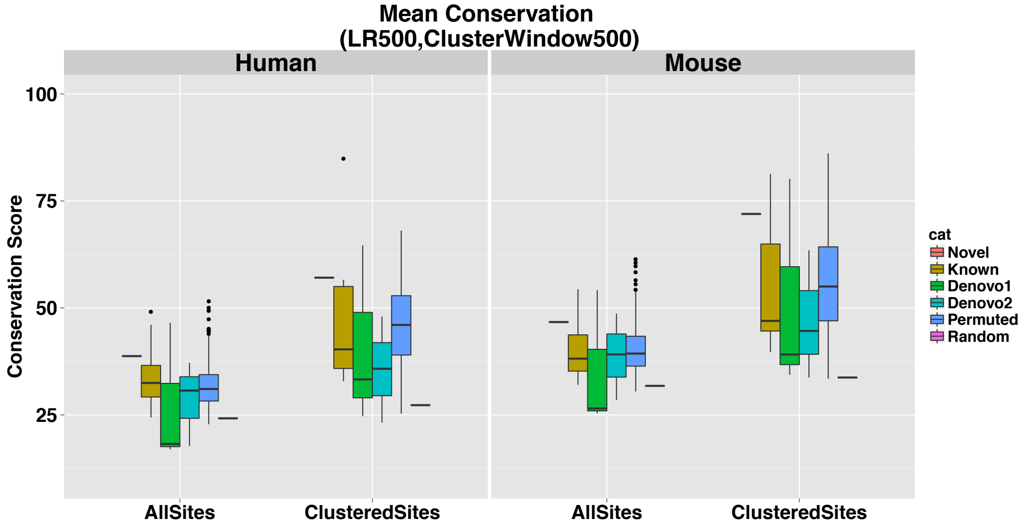

b LR = 2000

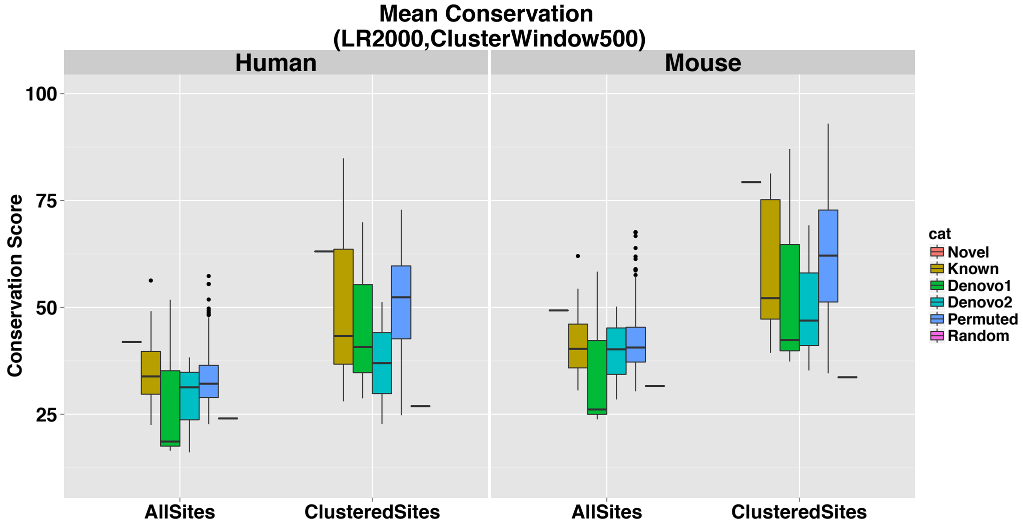

Supplementary Figure S12. Comparisons of phylogenetic conservation in human (left) and mouse (right) between different motif categories using different motif mapping criteria. (a) Likelihood ratio for mapping the new motif ≥ 500. (b) LR ≥ 2000. For each species, conservation was compared based on both all motif sites ("AllSites") and clustered motif sites ("ClusteredSites"). Within each motif site category, the boxplots from left to right show the overall conservation of the novel motif ("Novel"), eight known motifs ("Known"), three *de novo* class 1 motifs ("Denovo1"), three *de novo* class 2 motifs ("Denovo2"), 500 permuted motifs ("Permuted"), and random genomic controls ("Random"). For each motif, the overall conservation was the average phastCons score across all motif sites. For each control motif type, the boxplot shows the distribution of overall conservation across all motifs. Motif site cluster is defined using 500bp window.

a Cluster window size = 250

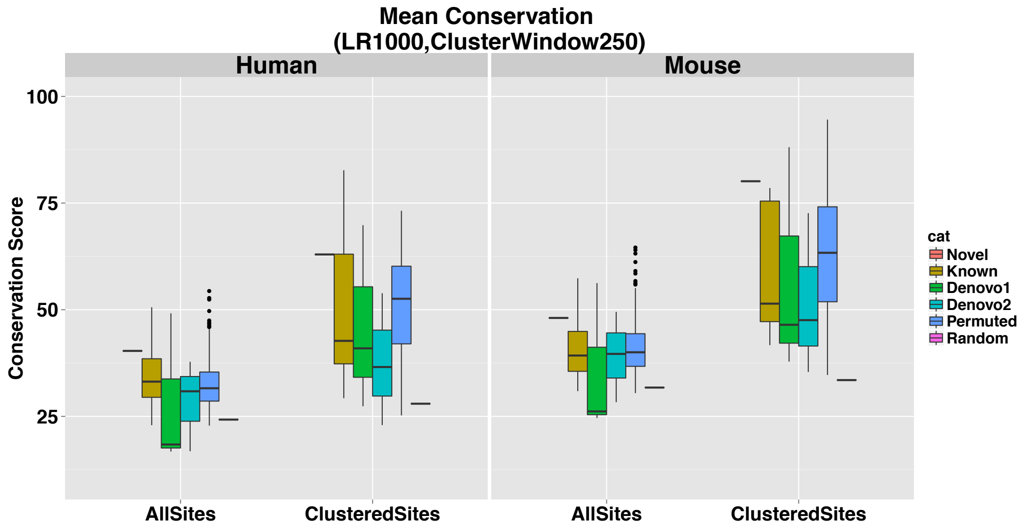

b Cluster window size = 1000

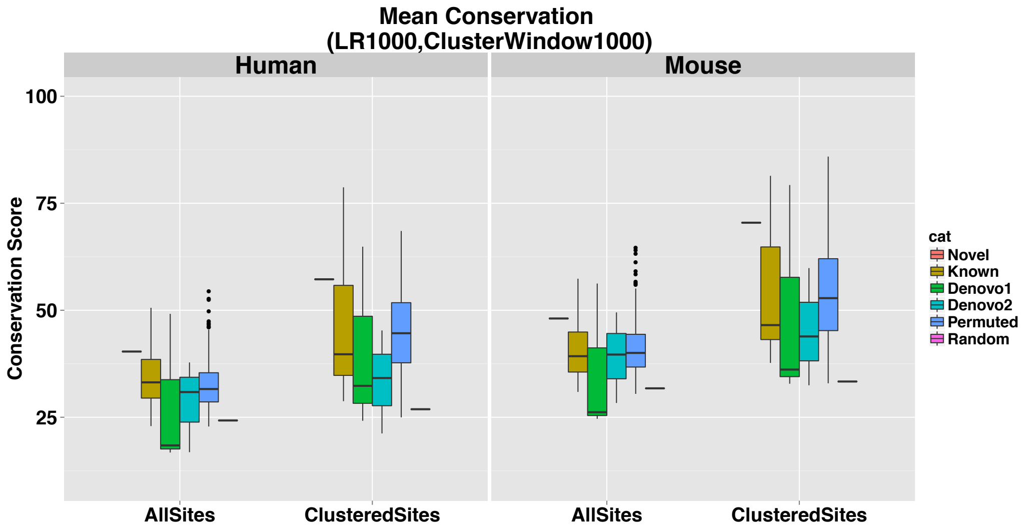

Supplementary Figure S13. Comparisons of phylogenetic conservation in human (left) and mouse (right) between different motif categories using different window size to define motif site clusters. (a) Windose size = 250bp. (b) Window size = 1000bp. For each species, conservation was compared based on both all motif sites ("AllSites") and clustered motif sites ("ClusteredSites"). Within each motif site category, the boxplots from left to right show the overall conservation of the novel motif ("Novel"), eight known motifs ("Known"), three *de novo* class 1 motifs ("Denovo1"), three *de novo* class 2 motifs ("Denovo2"), 500 permuted motifs ("Permuted"), and random genomic controls ("Random"). For each motif, the overall conservation was the average phastCons score across all motif sites. For each control motif type, the boxplot shows the distribution of overall conservation across all motifs. Motif mapping likelihood ratio ≥ 1000 for the new motif.

a LR = 500

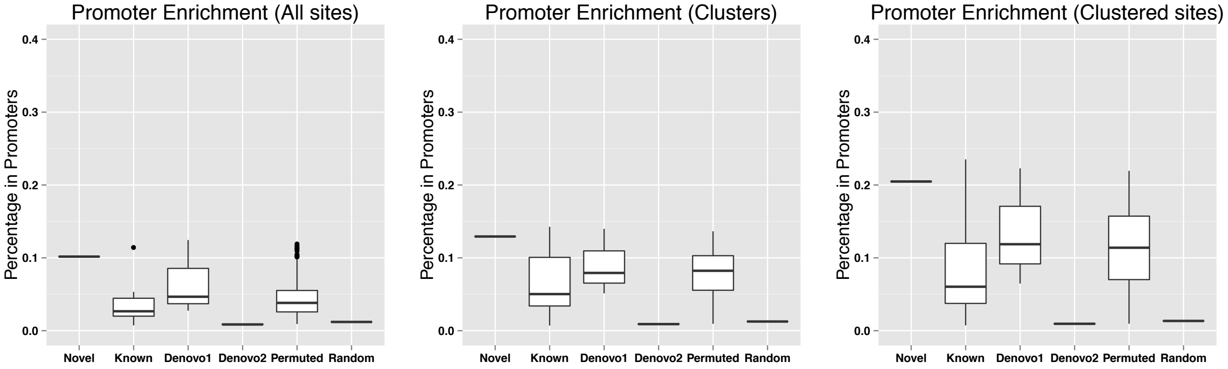

b LR = 2000

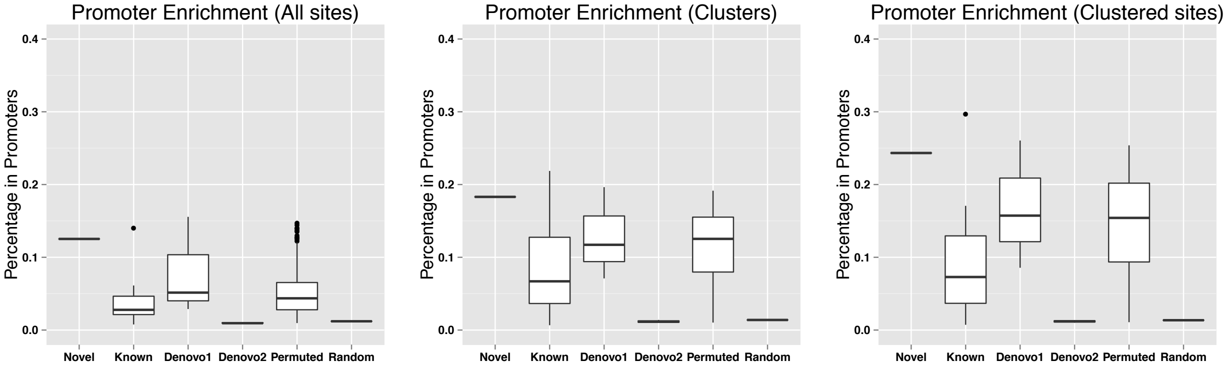

Supplementary Figure S14. Human promoter enrichment analysis performed using different motif mapping criteria. (a) Motif mapping likelihood ratio ≥ 500 for the new motif. (b) Likelihood ratio ≥ 2000 for the new motif. The percentages of motif sites (left), motif site clusters (middle), and clustered motif sites (right) that are in promoter regions are computed for each motif. Distributions of these percentages across all motifs are shown for the novel motif ("Novel"), eight known motifs ("Known"), three *de novo* class 1 motifs ("Denovo1"), three *de novo* class 2 motifs ("Denovo2"), 500 permuted motifs ("Permuted"), and random genomic control sites ("Random"). Promoter is defined as 1kb upstream of TSS. Motif site cluster is defined using 500bp window.

a LR = 500

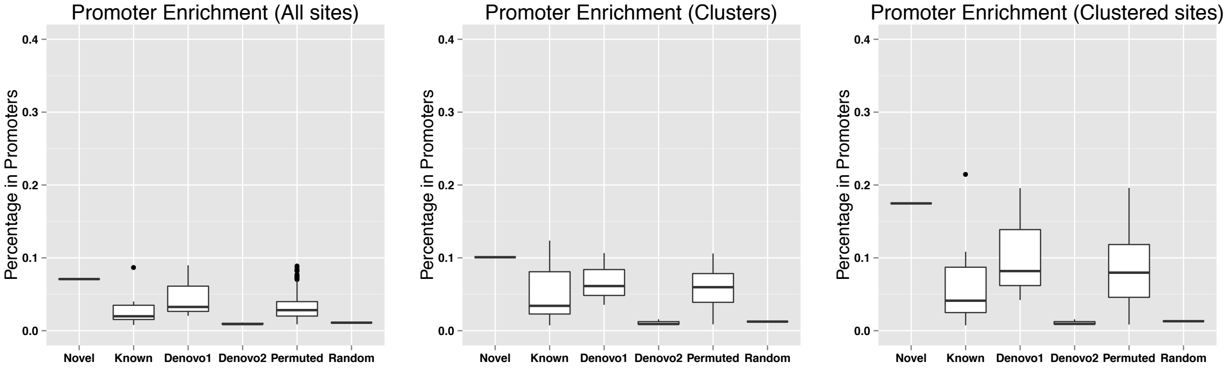

b LR = 2000

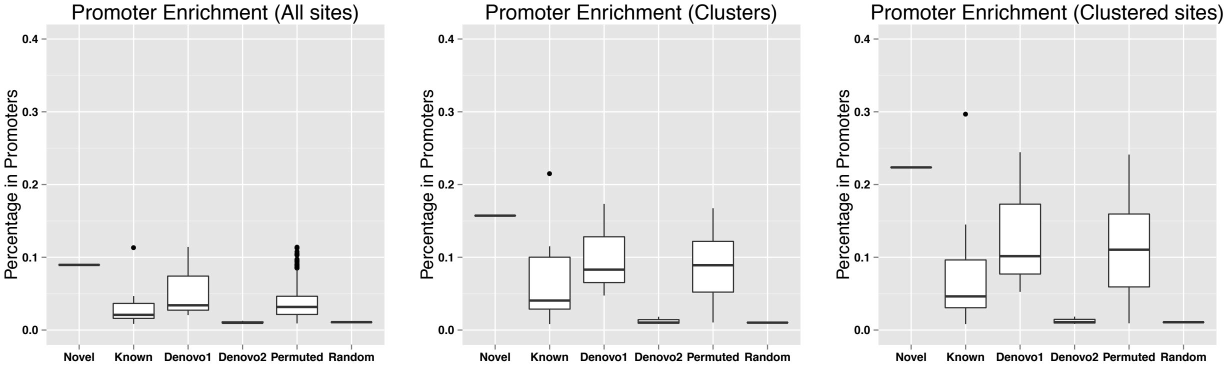

Supplementary Figure S15. Mouse promoter enrichment analysis performed using different motif mapping criteria. (a) Motif mapping likelihood ratio ≥ 500 for the new motif. (b) Likelihood ratio ≥ 2000 for the new motif. The percentages of motif sites (left), motif site clusters (middle), and clustered motif sites (right) that are in promoter regions are computed for each motif. Distributions of these percentages across all motifs are shown for the novel motif (“Novel”), eight known motifs (“Known”), three *de novo* class 1 motifs (“Denovo1”), three *de novo* class 2 motifs (“Denovo2”), 500 permuted motifs (“Permuted”), and random genomic control sites (“Random”). Promoter is defined as 1kb upstream of TSS. Motif site cluster is defined using 500bp window.

a Cluster window size = 250

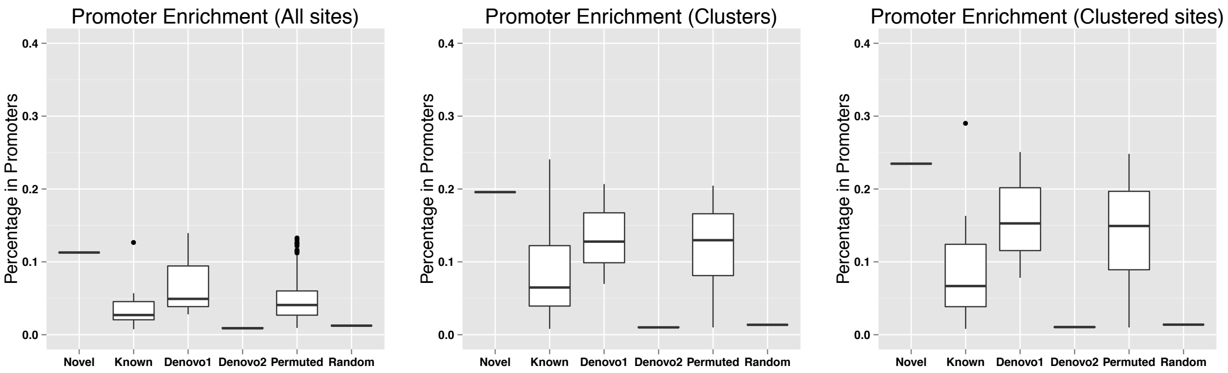

b Cluster window size =1000

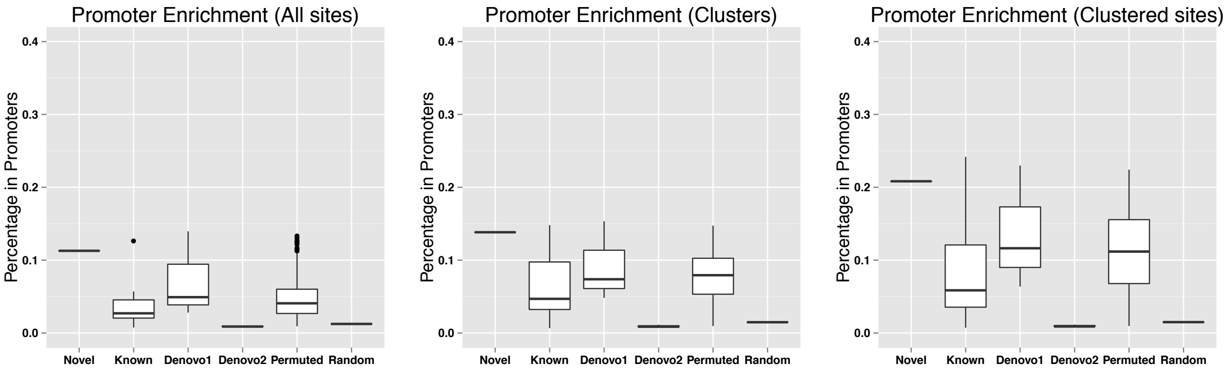

Supplementary Figure S16. Human promoter enrichment analysis performed using different window size to define motif site clusters. (a) Window size = 250bp; (b) Window size = 1000bp. The percentages of motif sites (left), motif site clusters (middle), and clustered motif sites (right) that are in promoter regions are computed for each motif. Distributions of these percentages across all motifs are shown for the novel motif ("Novel"), eight known motifs ("Known"), three *de novo* class 1 motifs ("Denovo1"), three *de novo* class 2 motifs ("Denovo2"), 500 permuted motifs ("Permuted"), and random genomic control sites ("Random"). Motif mapping likelihood ratio ≥ 1000 for the new motif. Promoter is defined as 1kb upstream of TSS.

a Cluster window size = 250

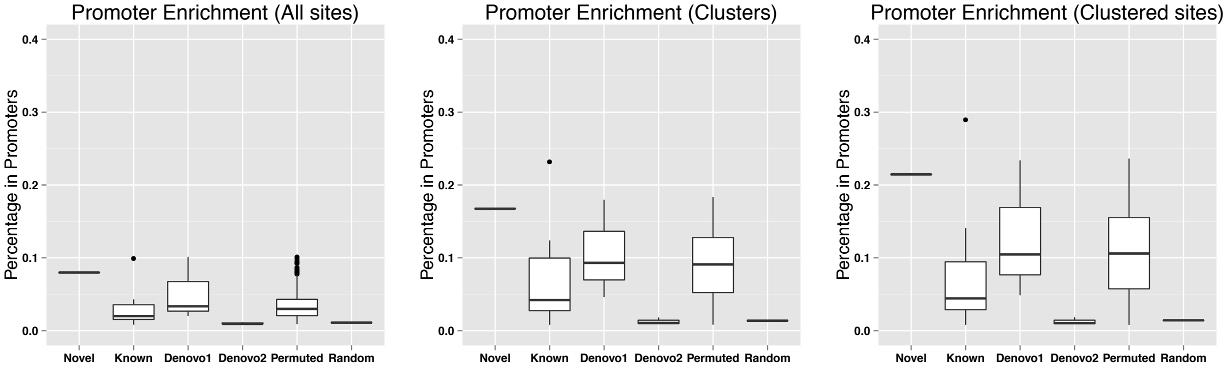

b Cluster window size =1000

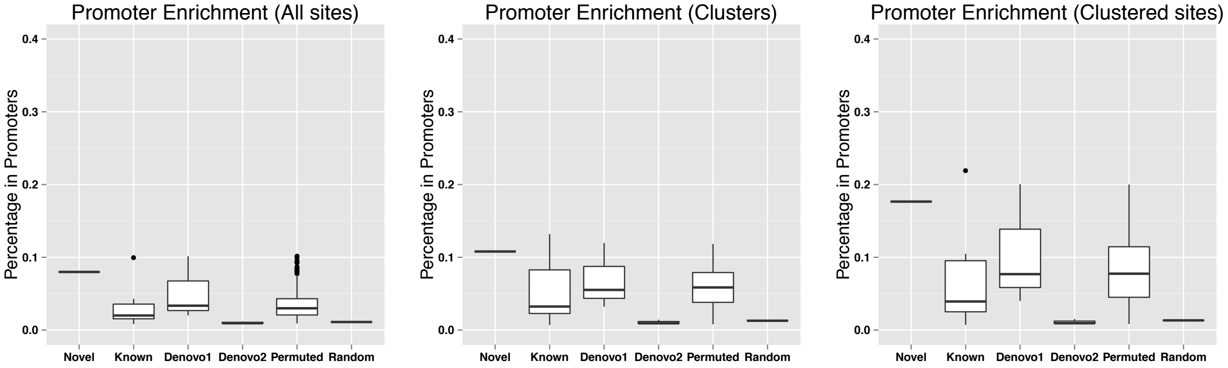

Supplementary Figure S17. Mouse promoter enrichment analysis performed using different window size to define motif site clusters. (a) Window size = 250bp; (b) Window size = 1000bp. The percentages of motif sites (left), motif site clusters (middle), and clustered motif sites (right) that are in promoter regions are computed for each motif. Distributions of these percentages across all motifs are shown for the novel motif ("Novel"), eight known motifs ("Known"), three *de novo* class 1 motifs ("Denovo1"), three *de novo* class 2 motifs ("Denovo2"), 500 permuted motifs ("Permuted"), and random genomic control sites ("Random"). Motif mapping likelihood ratio ≥ 1000 for the new motif. Promoter is defined as 1kb upstream of TSS.

a TSS up = 0.5kb

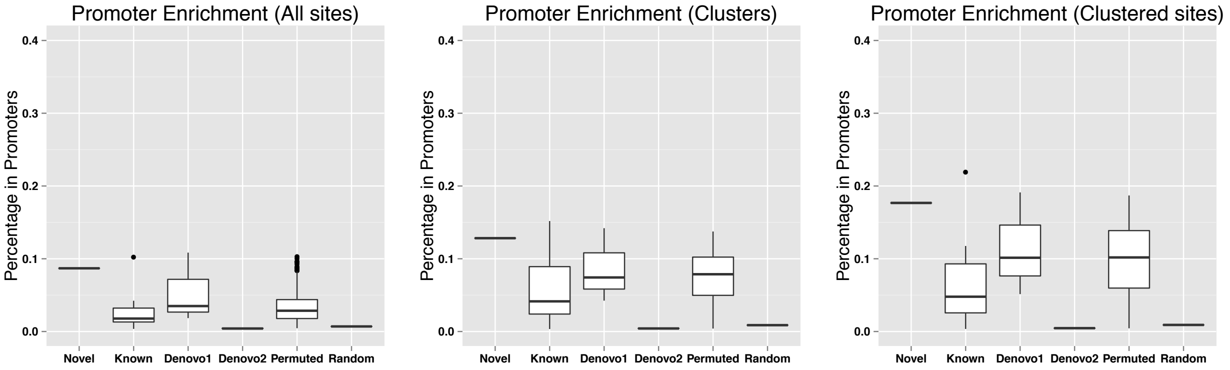

b TSS up = 1.5kb

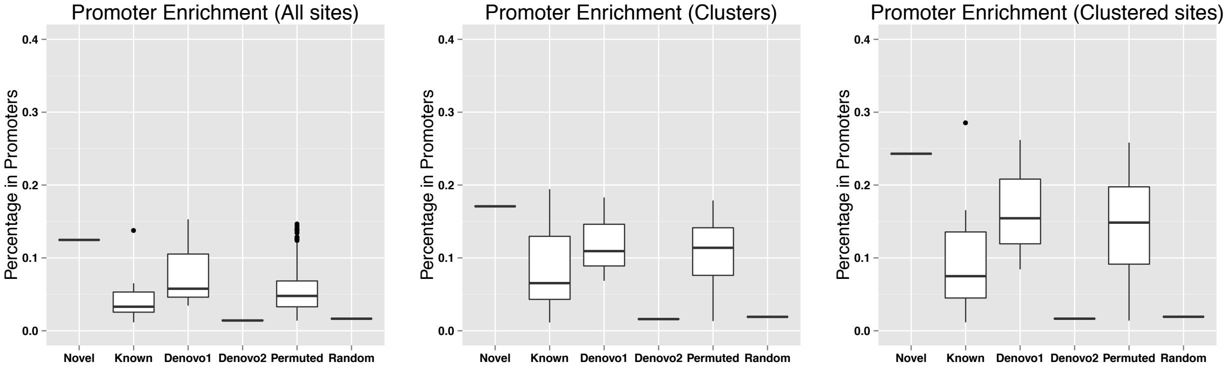

c TSS up = 2kb

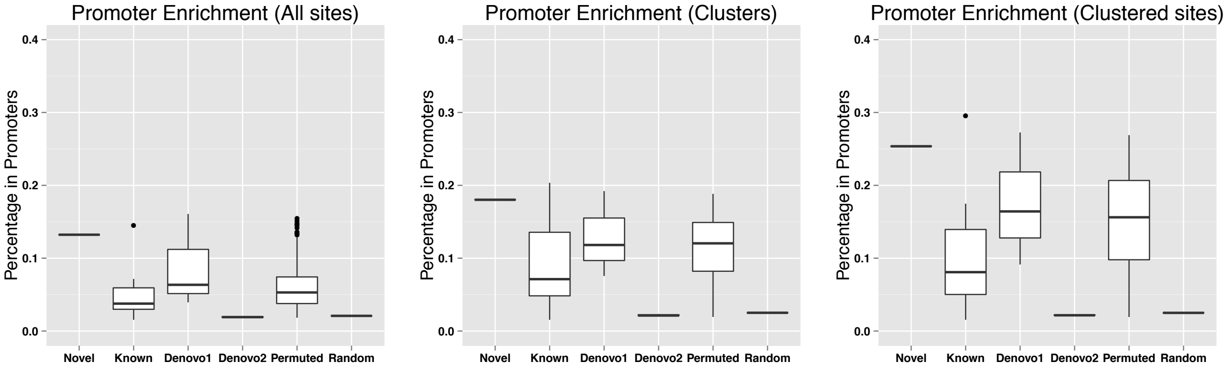

Supplementary Figure S18. Human promoter enrichment analysis performed using different promoter definitions. Promoter is defined using (a) 0.5kb upstream of TSS; (b) 1.5kb upstream of TSS; (c) 2kb upstream of TSS. The percentages of motif sites (left), motif site clusters (middle), and clustered motif sites (right) that are in promoter regions are computed for each motif. Distributions of these percentages across all motifs are shown for the novel motif ("Novel"), eight known motifs ("Known"), three *de novo* class 1 motifs ("Denovo1"), three *de novo* class 2 motifs ("Denovo2"), 500 permuted motifs ("Permuted"), and random genomic control sites ("Random"). Motif mapping likelihood ratio ≥ 1000 for the new motif. Motif site cluster is defined using 500bp window.

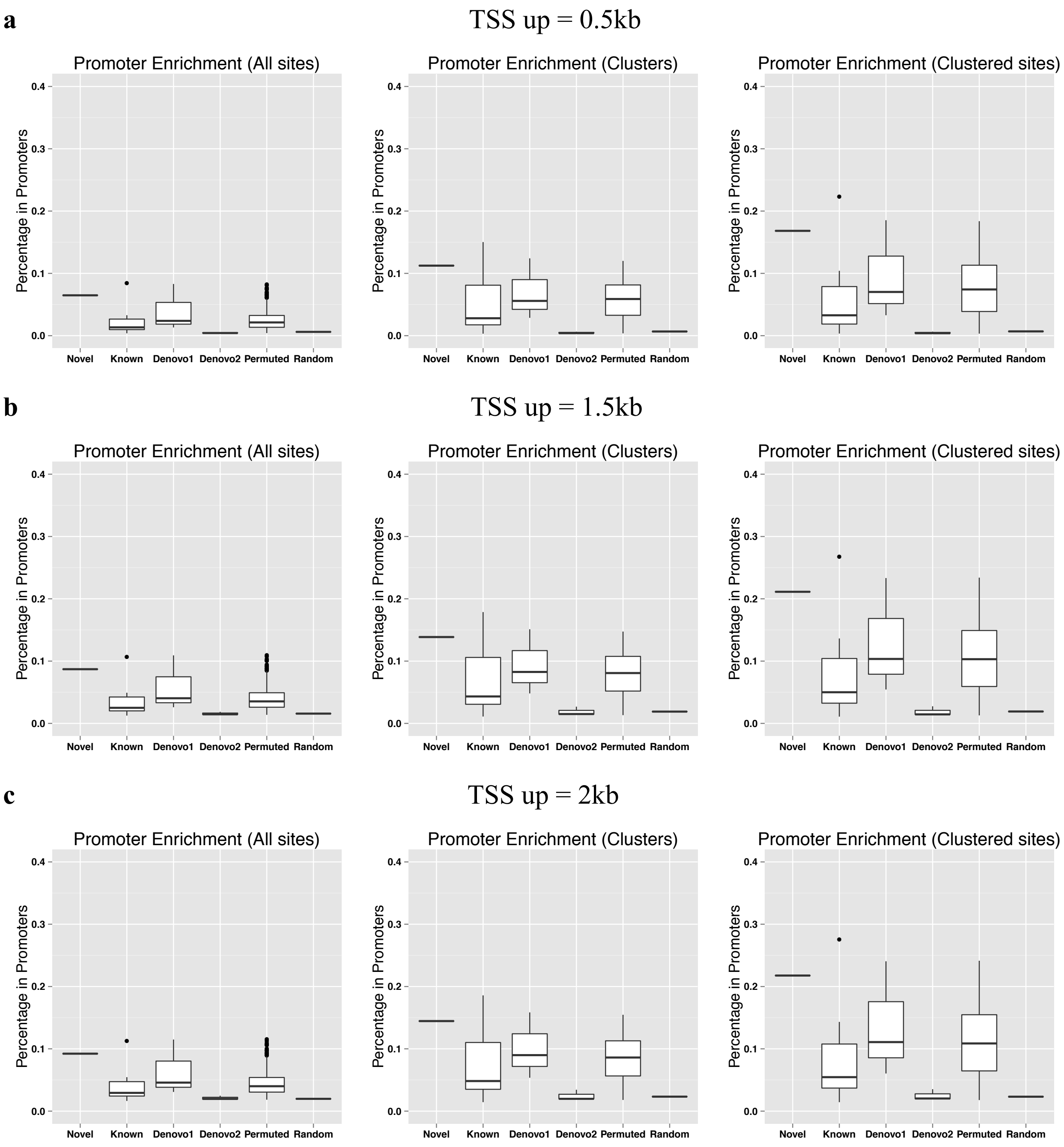

Supplementary Figure S19. Mouse promoter enrichment analysis performed using different promoter definitions. Promoter is defined using (a) 0.5kb upstream of TSS; (b) 1.5kb upstream of TSS; (c) 2kb upstream of TSS. The percentages of motif sites (left), motif site clusters (middle), and clustered motif sites (right) that are in promoter regions are computed for each motif. Distributions of these percentages across all motifs are shown for the novel motif ("Novel"), eight known motifs ("Known"), three *de novo* class 1 motifs ("Denovo1"), three *de novo* class 2 motifs ("Denovo2"), 500 permuted motifs ("Permuted"), and random genomic control sites ("Random"). Motif mapping likelihood ratio ≥ 1000 for the new motif. Motif site cluster is defined using 500bp window.

a LR = 500

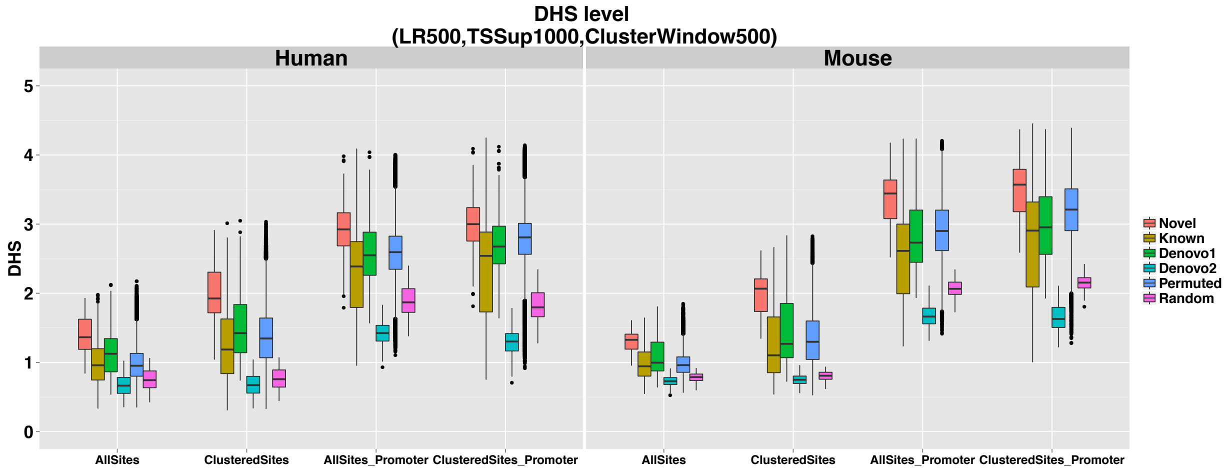

b LR = 2000

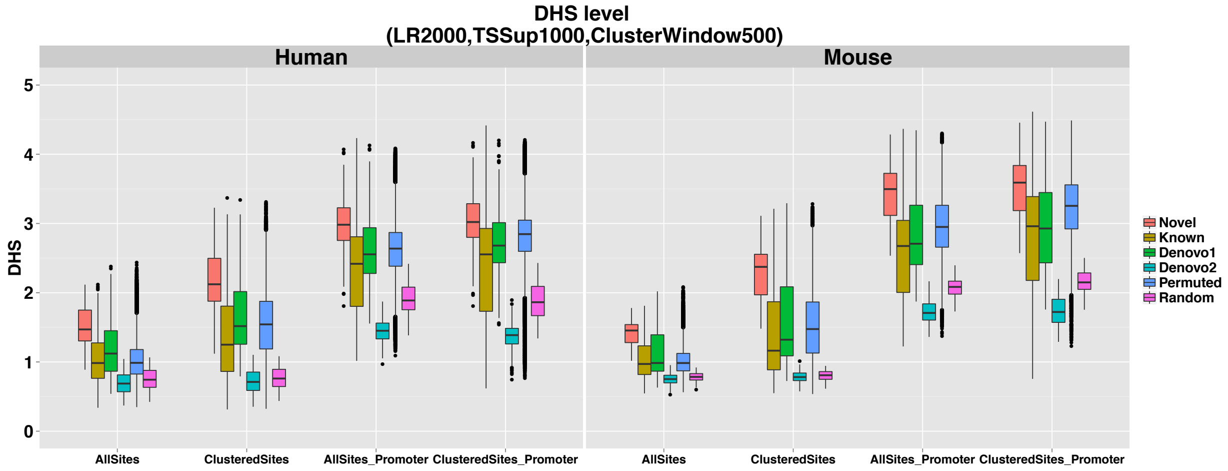

Supplementary Figure S20. Comparisons of DHS in human (left) and mouse (right) ENCODE cell lines between different motif categories using different motif mapping criteria. (a) Likelihood ratio for mapping new motif ≥ 500. (b) LR ≥ 2000. For each species, DHS was compared for four categories of motif sites: all motif sites ("AllSites"), clustered motif sites ("ClusteredSites"), all motif sites in promoters ("AllSites_Promoter"), and clustered motif sites in promoters ("ClusteredSites_Promoter"). Within each category, DHS was extracted for motif sites of the novel motif ("Novel"), eight known motifs ("Known"), three *de novo* class 1 motifs ("Denovo1"), three *de novo* class 2 motifs ("Denovo2"), 500 permuted motifs ("Permuted"), and random genomic controls ("Random"). For each motif, the average DHS across all motif sites was obtained. The distribution of this average DHS across all motifs and all cell lines in each motif category is then shown using boxplot. Promoter is defined as 1kb upstream of TSS. Motif site cluster is defined using 500bp window.

a Cluster window size = 250

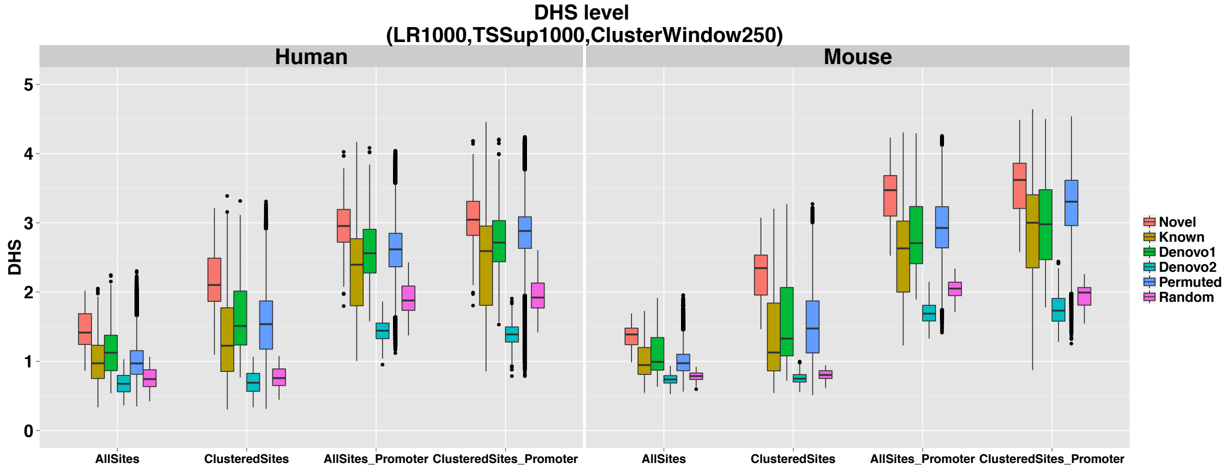

b Cluster window size = 1000

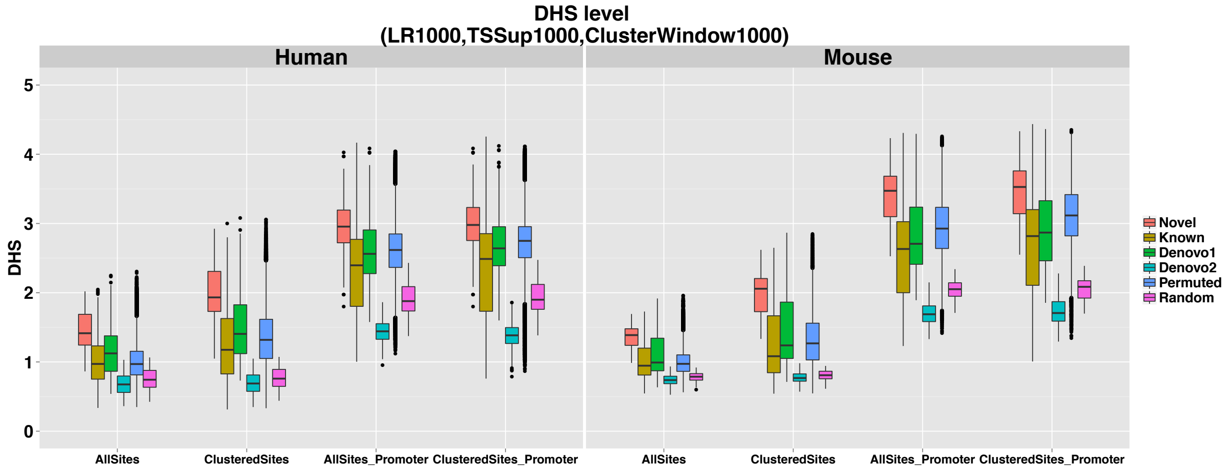

Supplementary Figure S21. Comparisons of DHS in human (left) and mouse (right) ENCODE cell lines between different motif categories using different window size to define motif site clusteres. (a) Window size = 250bp. (b) Window size = 1000bp. For each species, DHS was compared for four categories of motif sites: all motif sites ("AllSites"), clustered motif sites ("ClusteredSites"), all motif sites in promoters ("AllSites_Promoter"), and clustered motif sites in promoters ("ClusteredSites_Promoter"). Within each category, DHS was extracted for motif sites of the novel motif ("Novel"), eight known motifs ("Known"), three *de novo* class 1 motifs ("Denovo1"), three *de novo* class 2 motifs ("Denovo2"), 500 permuted motifs ("Permuted"), and random genomic controls ("Random"). For each motif, the average DHS across all motif sites was obtained. The distribution of this average DHS across all motifs and all cell lines in each motif category is then shown using boxplot. Motif mapping likelihood ratio ≥ 1000 for the new motif. Promoter is defined as 1kb upstream of TSS.

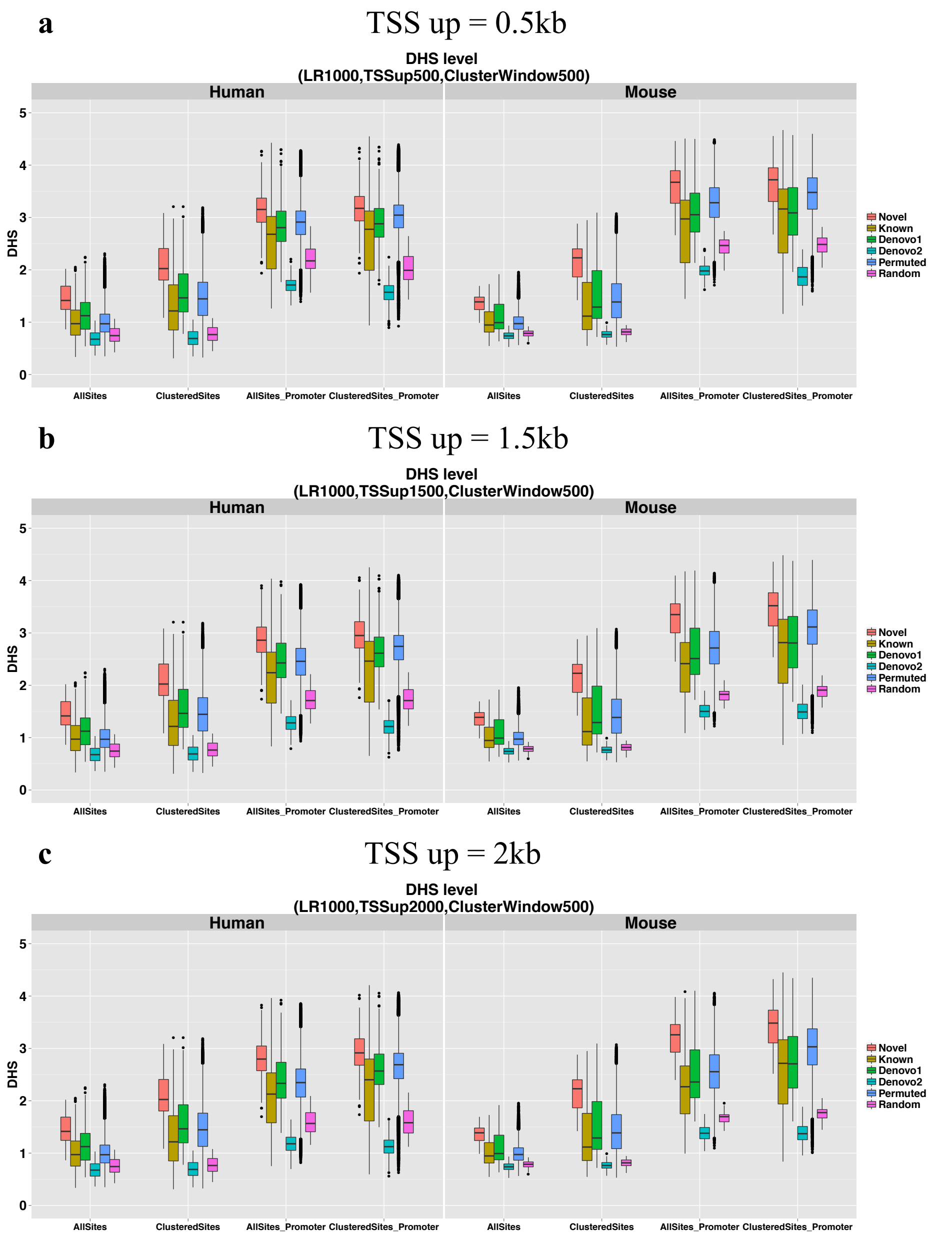

Supplementary Figure S22. Comparisons of DHS in human (left) and mouse (right) ENCODE cell lines between different motif categories using different promoter definitions. (a) 0.5kb upstream of TSS. (b) 1.5kb upstream of TSS. (c) 2kb upstream of TSS. For each species, DHS was compared for four categories of motif sites: all motif sites ("AllSites"), clustered motif sites ("ClusteredSites"), all motif sites in promoters ("AllSites_Promoter"), and clustered motif sites in promoters ("ClusteredSites_Promoter"). Within each category, DHS was extracted for motif sites of the novel motif ("Novel"), eight known motifs ("Known"), three *de novo* class 1 motifs ("Denovo1"), three *de novo* class 2 motifs ("Denovo2"), 500 permuted motifs ("Permuted"), and random genomic controls ("Random"). For each motif, the average DHS across all motif sites was obtained. The distribution of this average DHS across all motifs and all cell lines in each motif category is then shown using boxplot. Motif mapping likelihood ratio ≥ 1000 for the new motif. Motif site clustering window size = 500bp.

References

1. Li, L., Liang, Y. & Bass, R. L. GAPWM: a genetic algorithm method for optimizing a position weight matrix. *Bioinformatics* **23**, 1188-1194 (2007).

2. ENCODE Project Consortium. An integrated encyclopedia of DNA elements in the human genome. *Nature* **489**, 57–74 (2012).

3. Mouse ENCODE Consortium et al. An encyclopedia of mouse DNA elements (Mouse ENCODE). *Genome Biol.* **13**, 418 (2012).
